# Supplementary material for: Preparing Doctors in Training for Health Activist Roles: A Cross-Institutional Community Organizing Workshop for Incoming Medical Residents
Source: MedEdPORTAL. 2022 Jan 18;18:11208. doi: 10.15766/mep_2374-8265.11208 (PMC8763867; doi:10.15766/mep_2374-8265.11208)
Supplement: Supplementary file 1 — Introduction to Community Organizing.pptxIntroduction to Public Narrative.pptxPredrag Stojicic Video.mp4Facilitator Manual.docxStory of Self Small-Group Guide.docxPostworkshop Survey.docx [file mep_2374-8265.11208-s001.zip › A. Introduction to Community Organizing.pptx]

## Slide 1
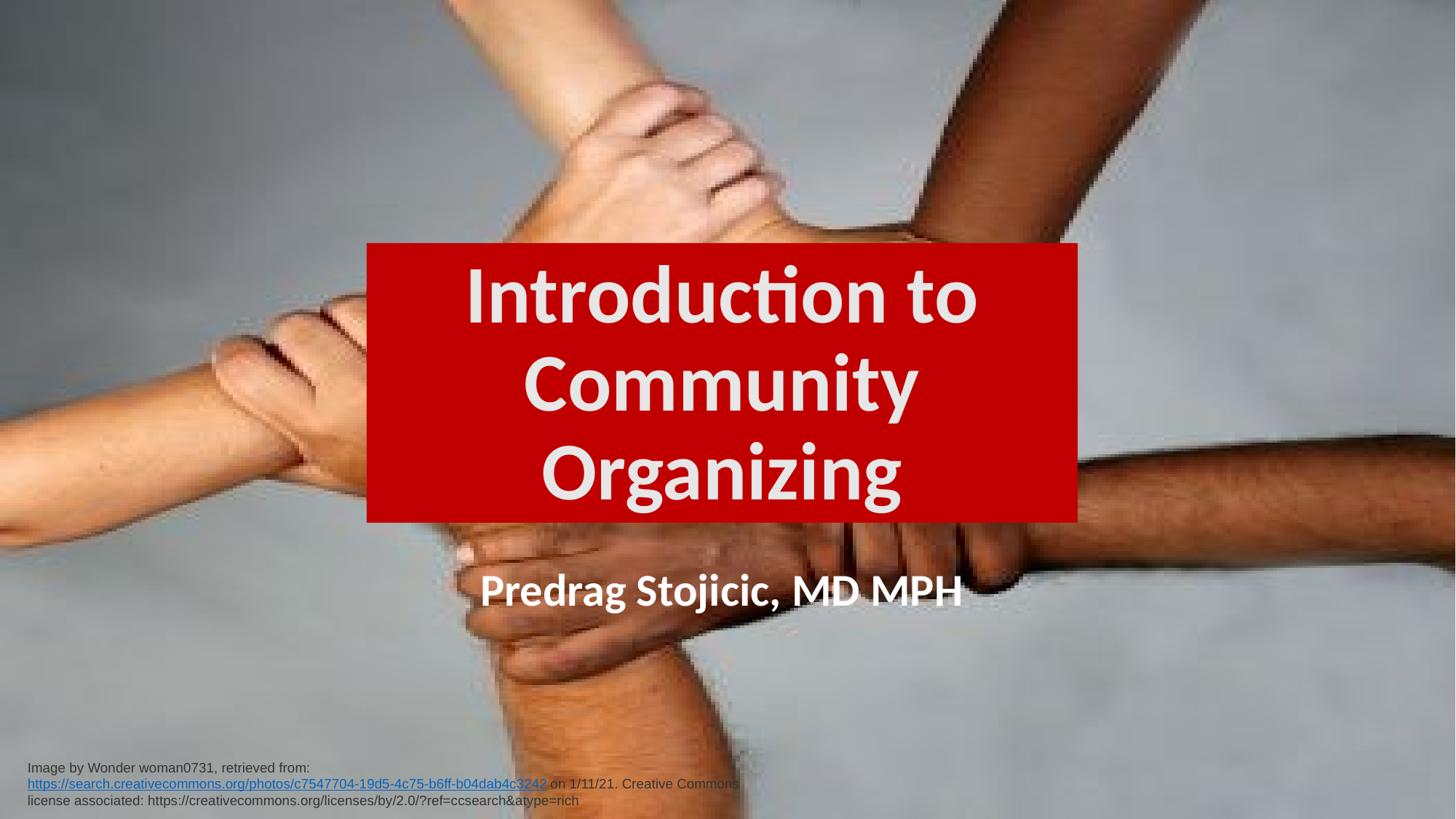

# Introduction to Community Organizing
Predrag Stojicic, MD MPH
Image by Wonder woman0731, retrieved from: https://search.creativecommons.org/photos/c7547704-19d5-4c75-b6ff-b04dab4c3242 on 1/11/21. Creative Commons license associated: https://creativecommons.org/licenses/by/2.0/?ref=ccsearch&atype=rich

## Slide 2
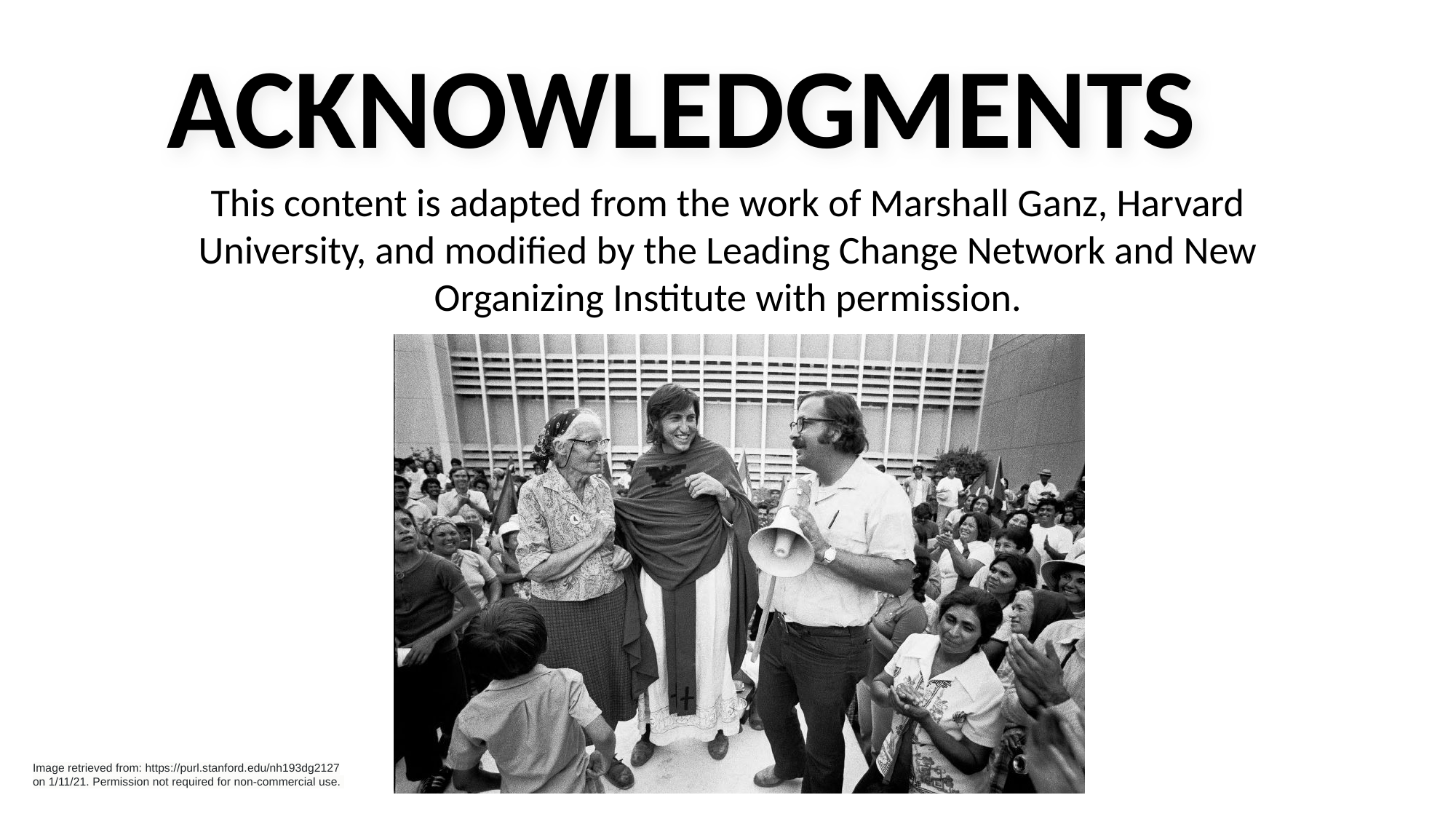

ACKNOWLEDGMENTS
This content is adapted from the work of Marshall Ganz, Harvard University, and modified by the Leading Change Network and New Organizing Institute with permission.
Image retrieved from: https://purl.stanford.edu/nh193dg2127
on 1/11/21. Permission not required for non-commercial use.

## Slide 3
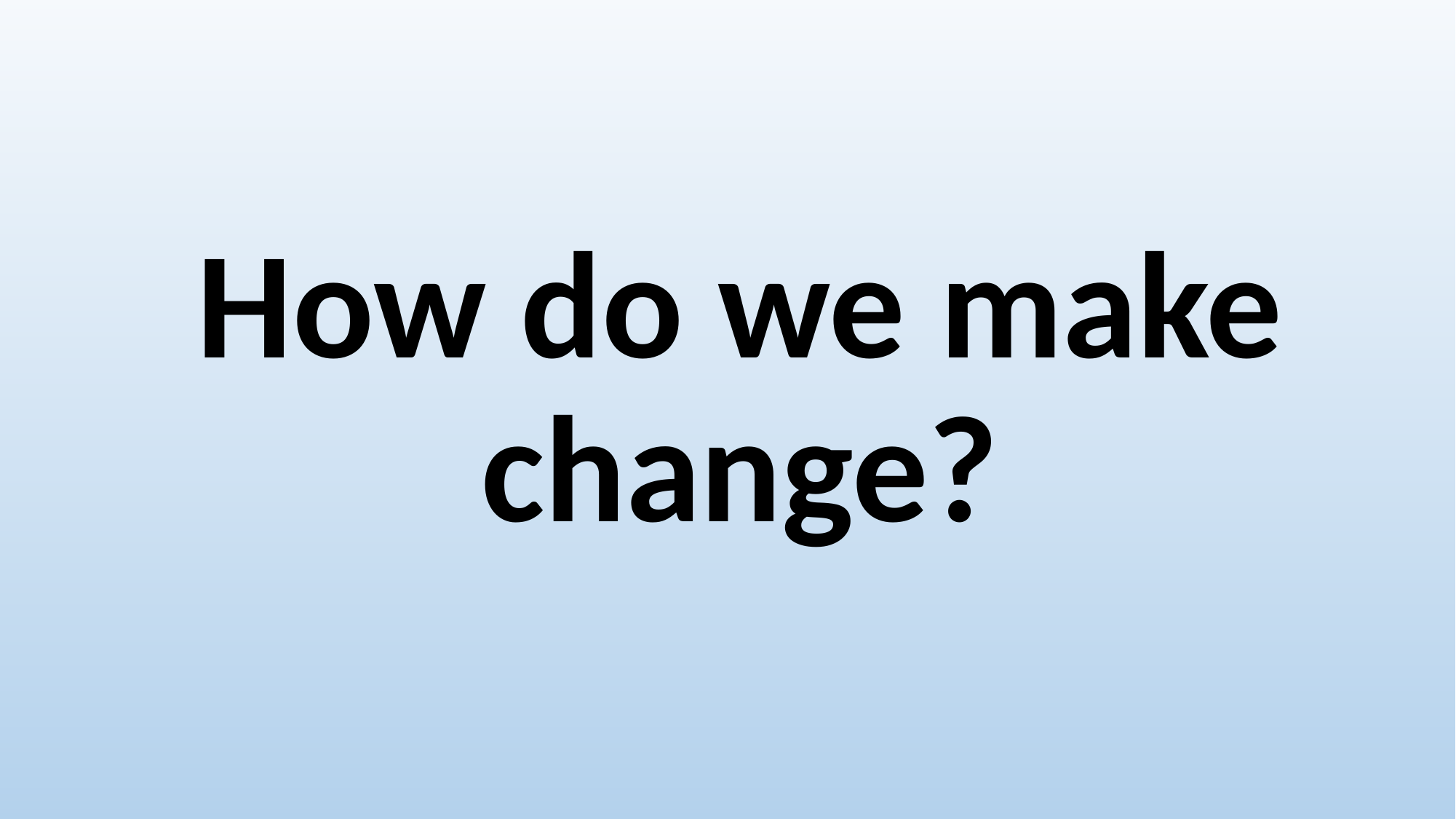

# How do we make change?

## Slide 4
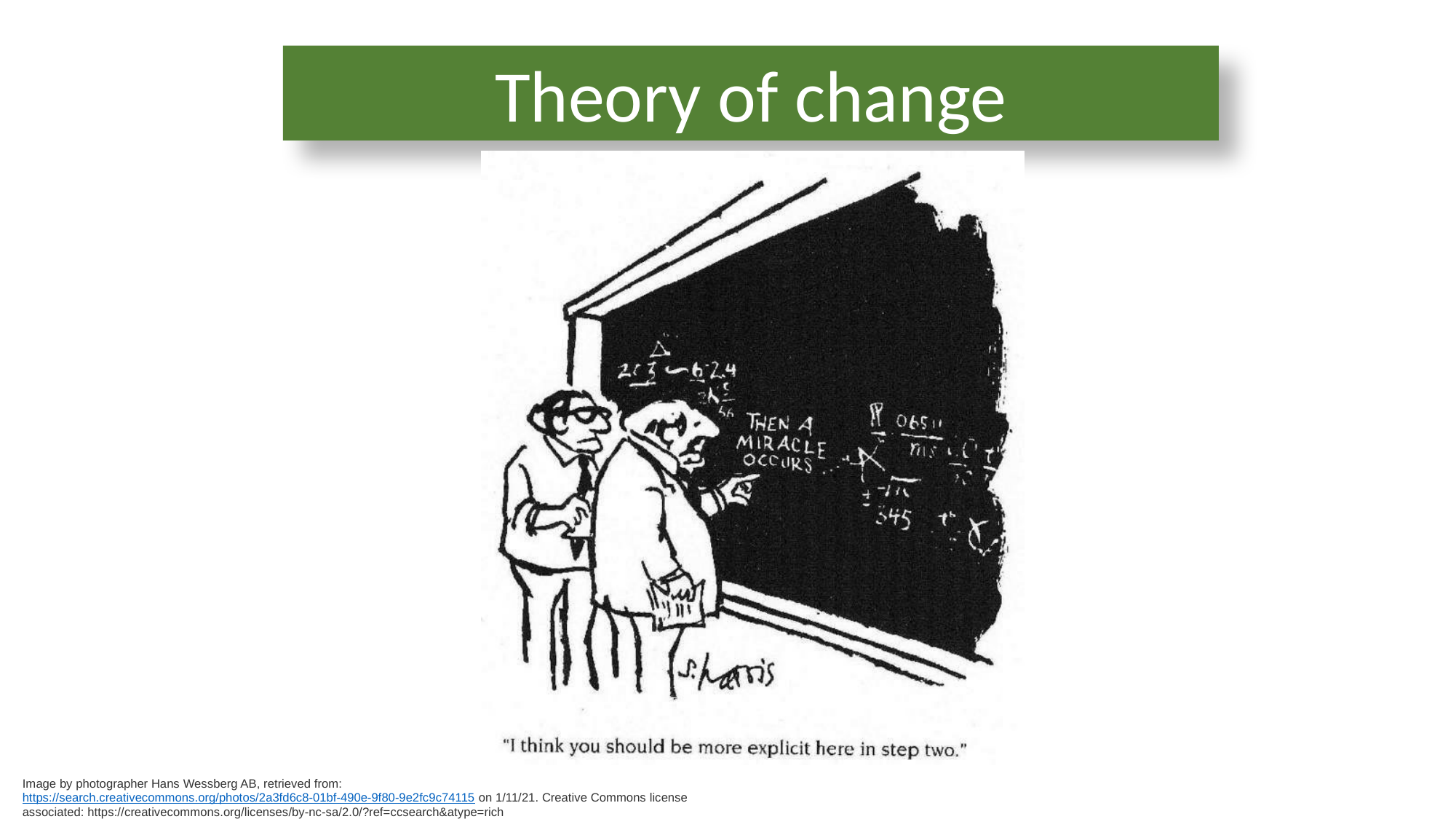

Theory of change
Image by photographer Hans Wessberg AB, retrieved from: https://search.creativecommons.org/photos/2a3fd6c8-01bf-490e-9f80-9e2fc9c74115 on 1/11/21. Creative Commons license associated: https://creativecommons.org/licenses/by-nc-sa/2.0/?ref=ccsearch&atype=rich

## Slide 5
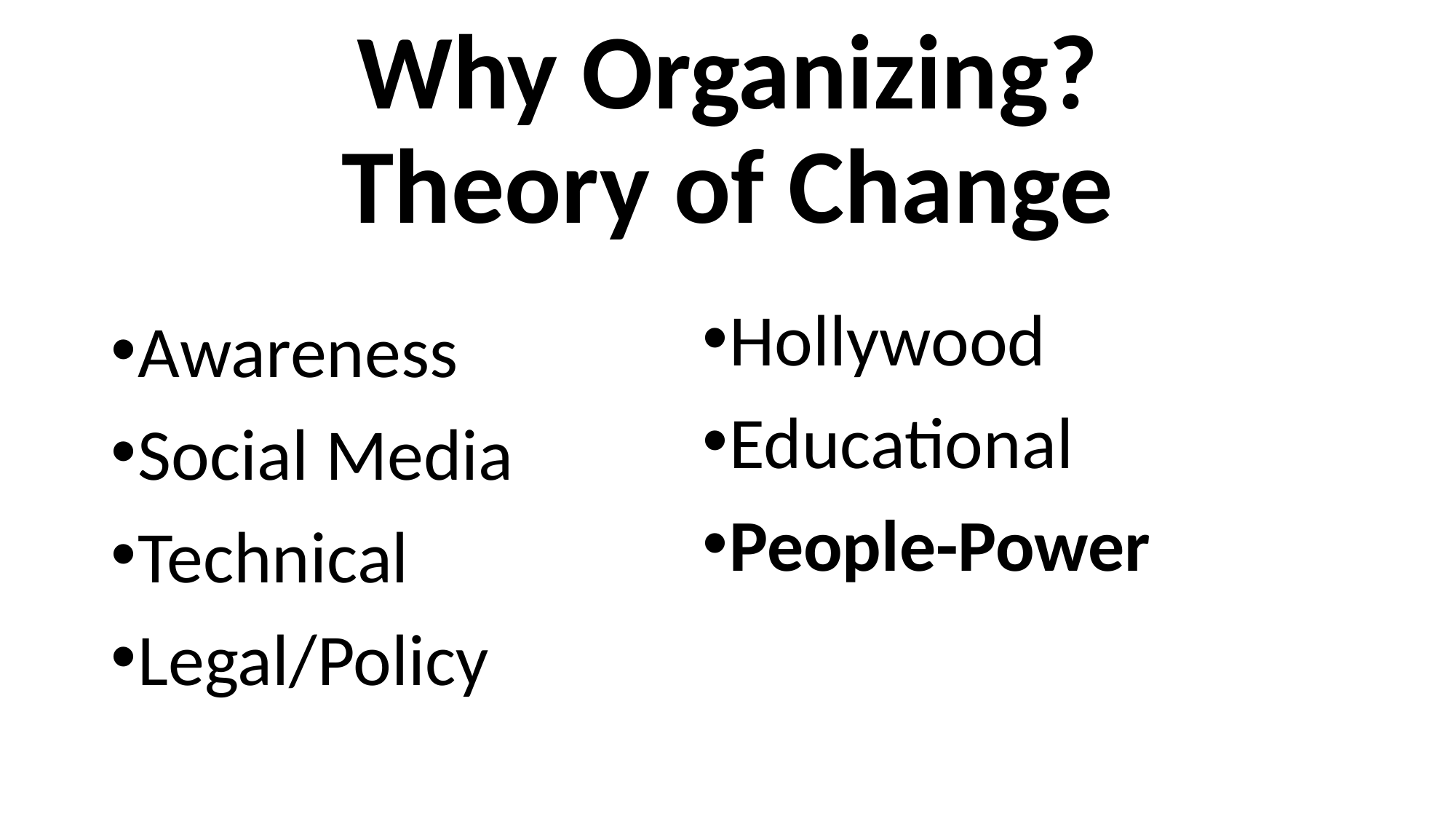

# Why Organizing?
Theory of Change
Hollywood
Educational
People-Power
Awareness
Social Media
Technical
Legal/Policy

## Slide 6
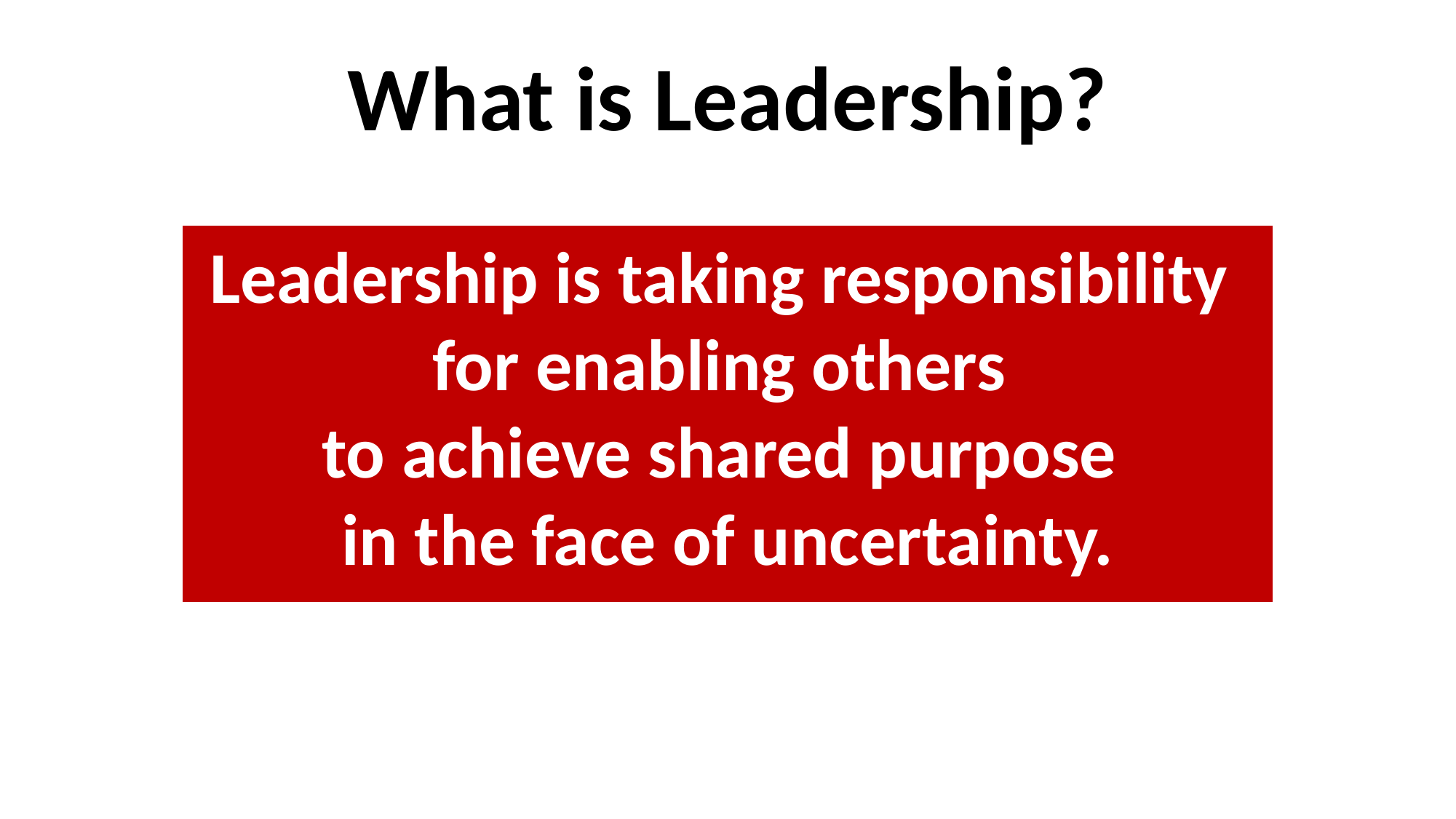

What is Leadership?
Leadership is taking responsibility
for enabling others
to achieve shared purpose
in the face of uncertainty.

## Slide 7
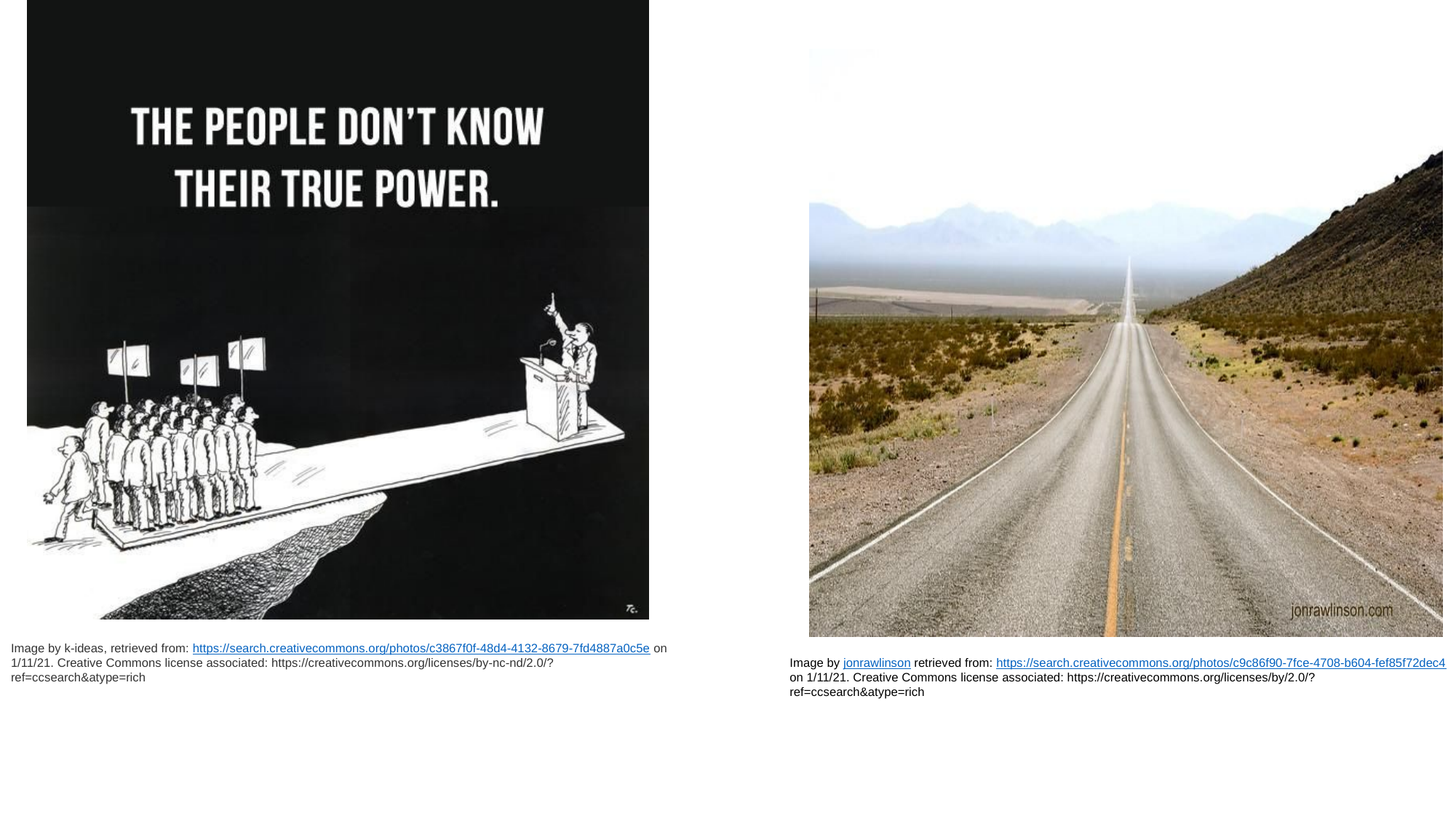

Image by k-ideas, retrieved from: https://search.creativecommons.org/photos/c3867f0f-48d4-4132-8679-7fd4887a0c5e on 1/11/21. Creative Commons license associated: https://creativecommons.org/licenses/by-nc-nd/2.0/?ref=ccsearch&atype=rich
Image by jonrawlinson retrieved from: https://search.creativecommons.org/photos/c9c86f90-7fce-4708-b604-fef85f72dec4 on 1/11/21. Creative Commons license associated: https://creativecommons.org/licenses/by/2.0/?ref=ccsearch&atype=rich

## Slide 8
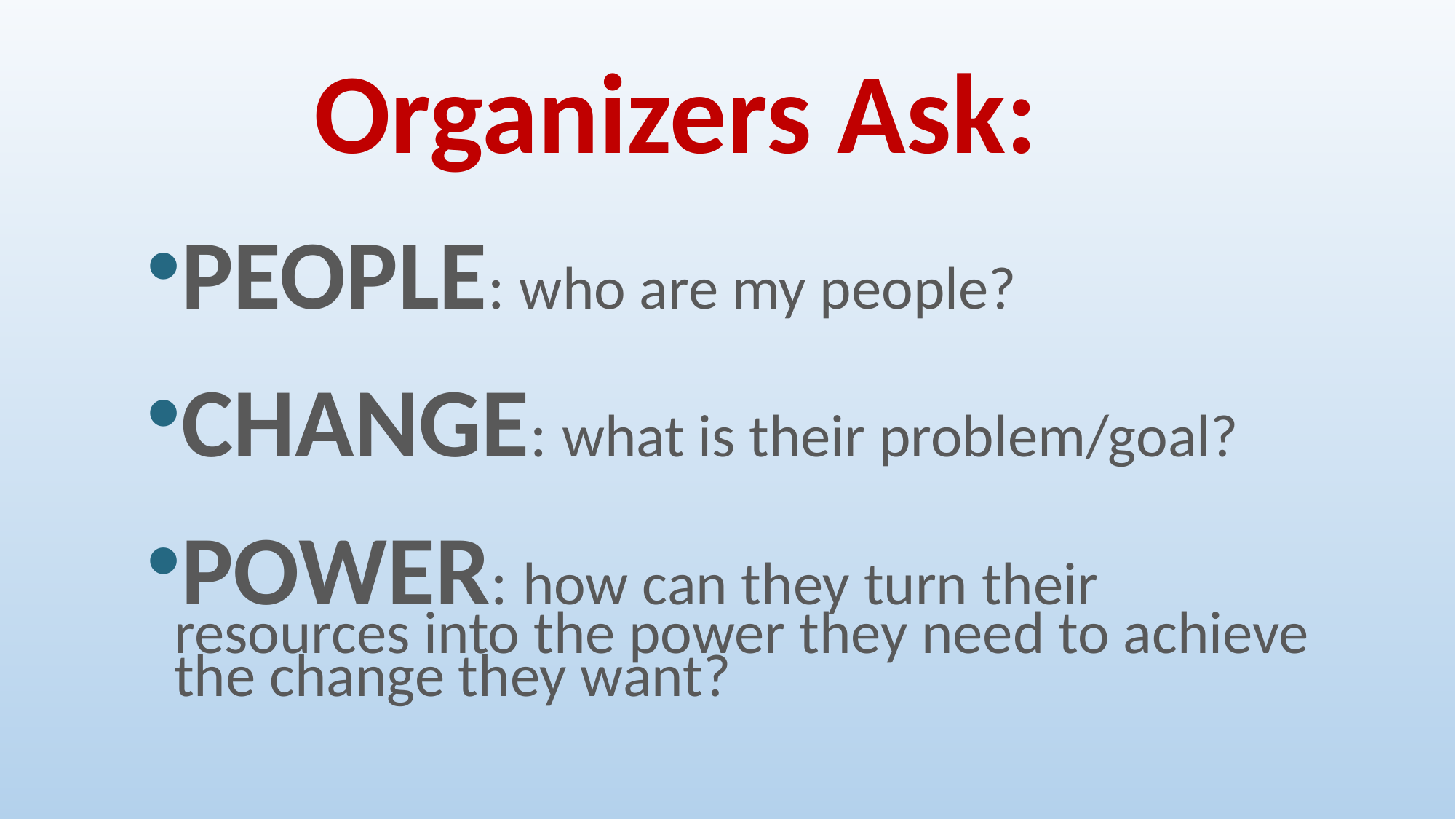

Organizers Ask:
PEOPLE: who are my people?
CHANGE: what is their problem/goal?
POWER: how can they turn their resources into the power they need to achieve the change they want?

## Slide 9
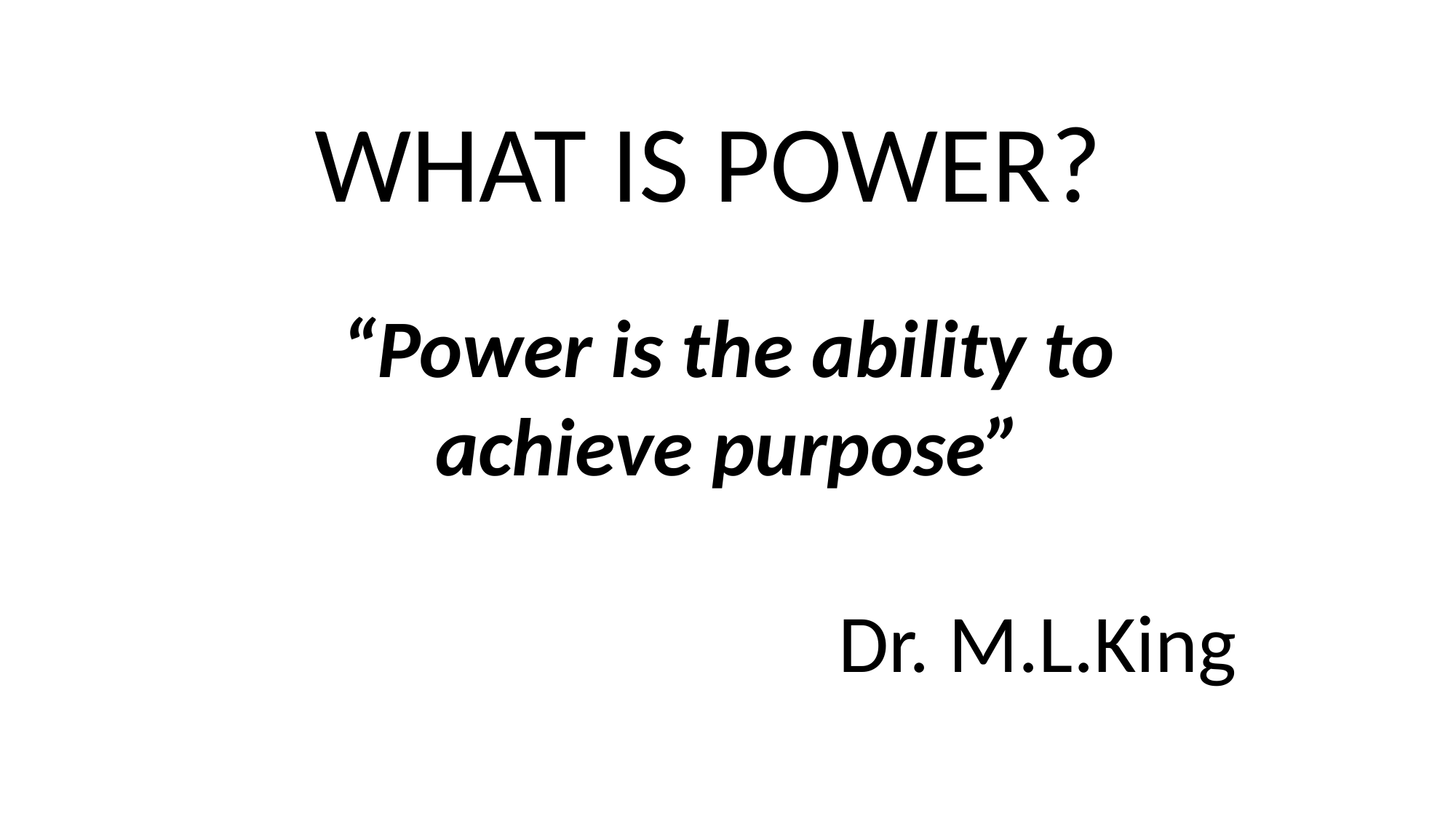

WHAT IS POWER?
“Power is the ability to achieve purpose”
Dr. M.L.King

## Slide 10
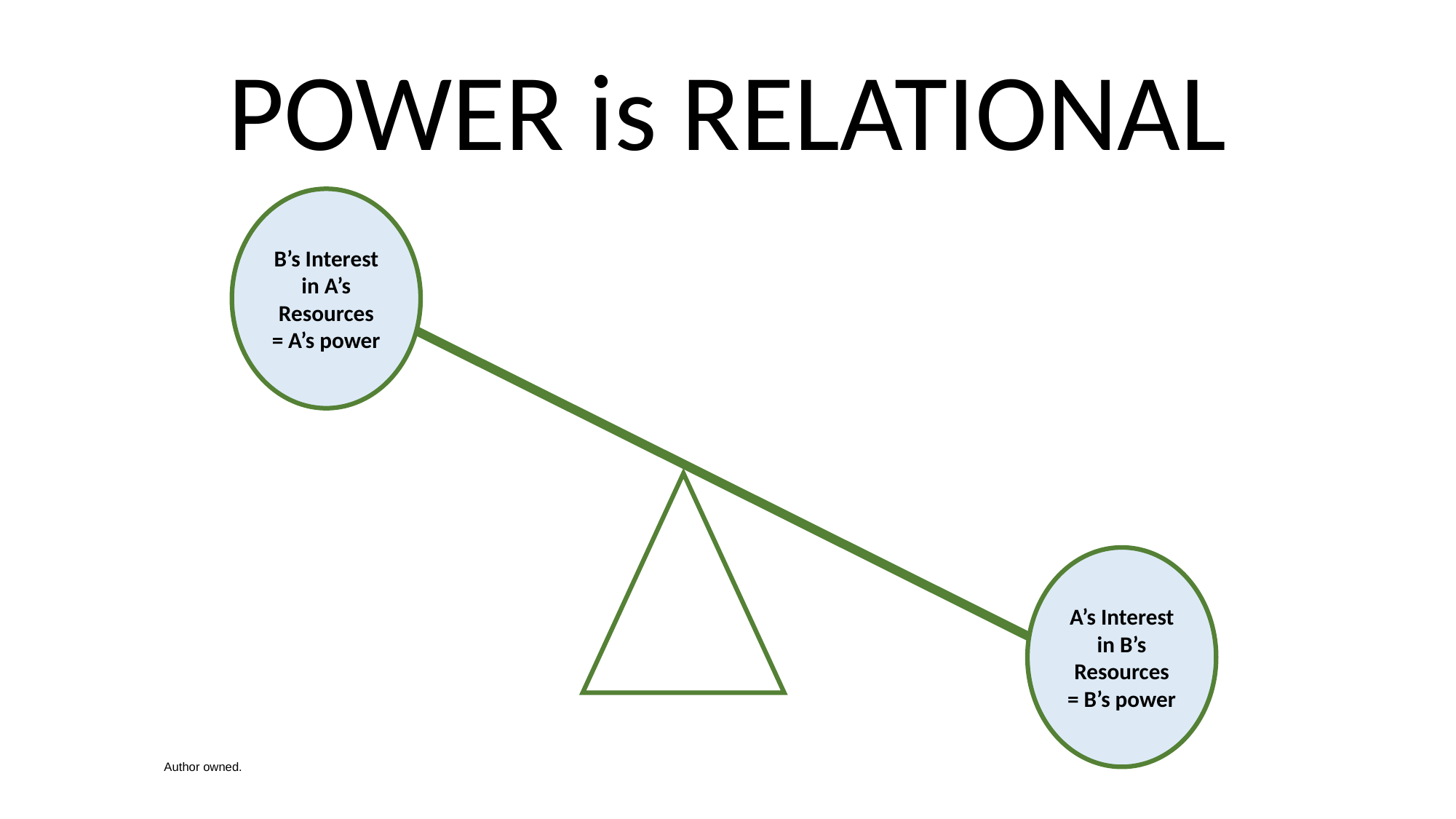

POWER is RELATIONAL
B’s Interest in A’s Resources = A’s power
A’s Interest in B’s Resources = B’s power
Author owned.

## Slide 11
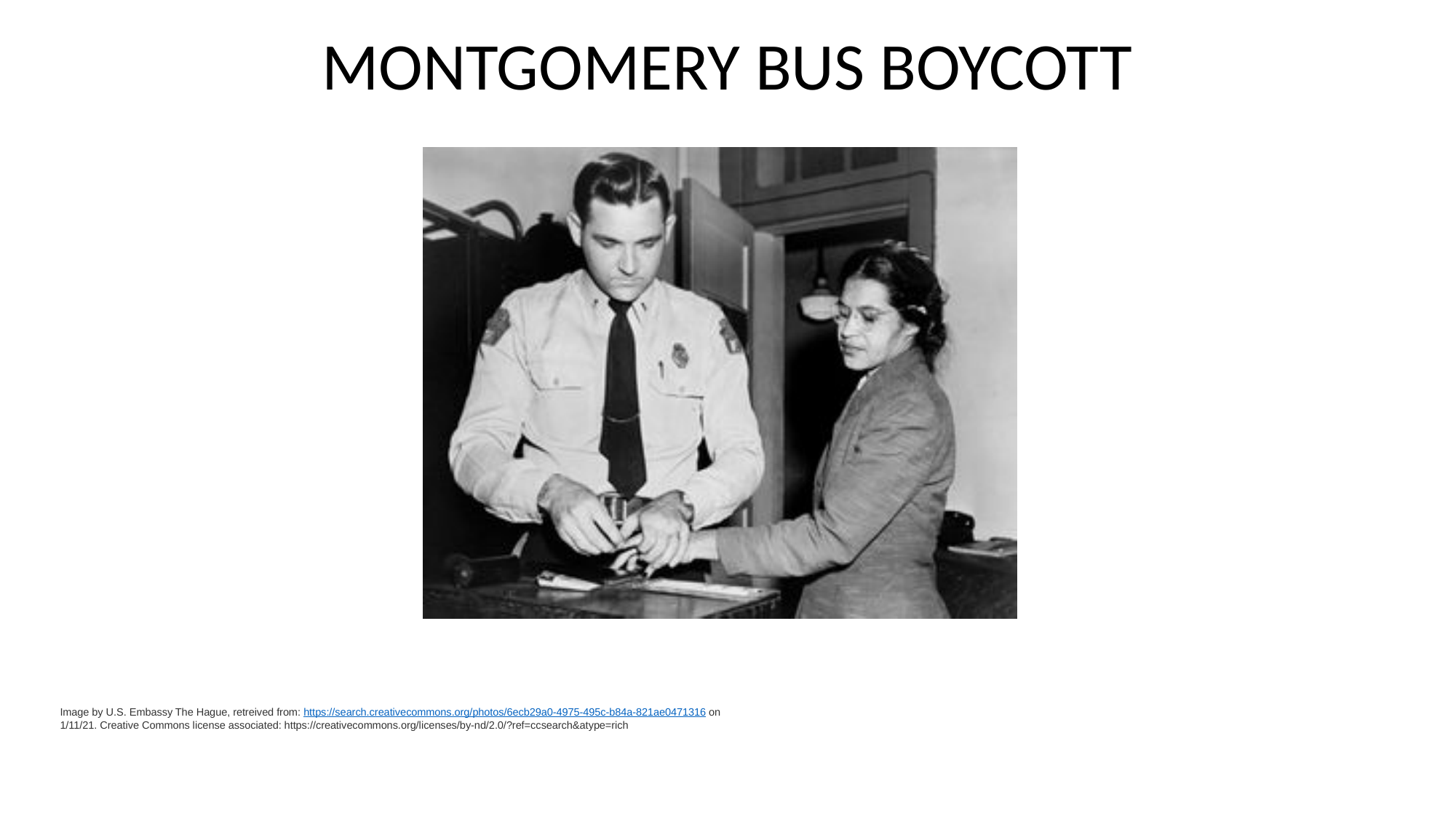

MONTGOMERY BUS BOYCOTT
Image by U.S. Embassy The Hague, retreived from: https://search.creativecommons.org/photos/6ecb29a0-4975-495c-b84a-821ae0471316 on 1/11/21. Creative Commons license associated: https://creativecommons.org/licenses/by-nd/2.0/?ref=ccsearch&atype=rich

## Slide 12
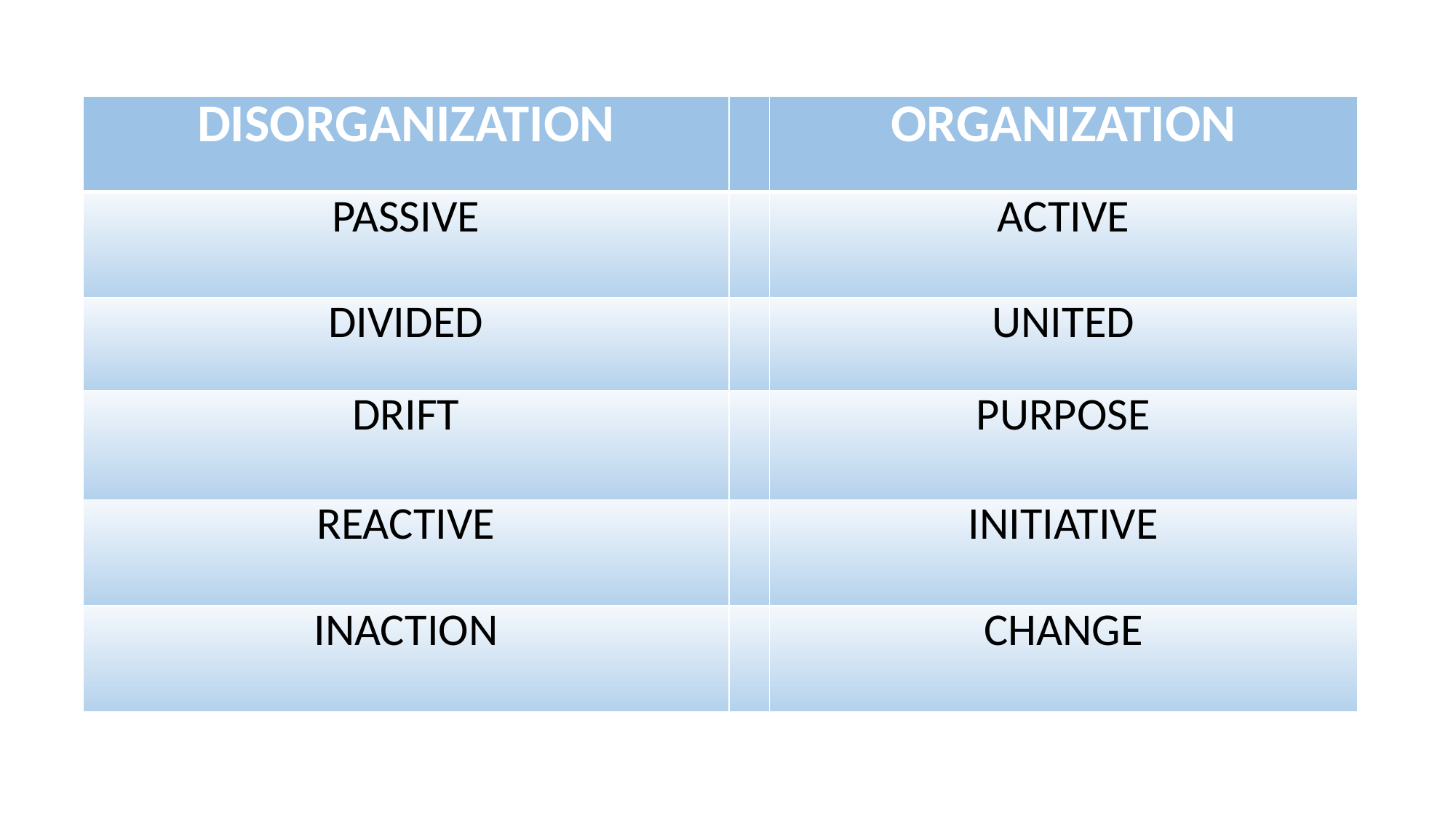

| DISORGANIZATION | | ORGANIZATION |
| --- | --- | --- |
| PASSIVE | | ACTIVE |
| DIVIDED | | UNITED |
| DRIFT | | PURPOSE |
| REACTIVE | | INITIATIVE |
| INACTION | | CHANGE |

## Slide 13
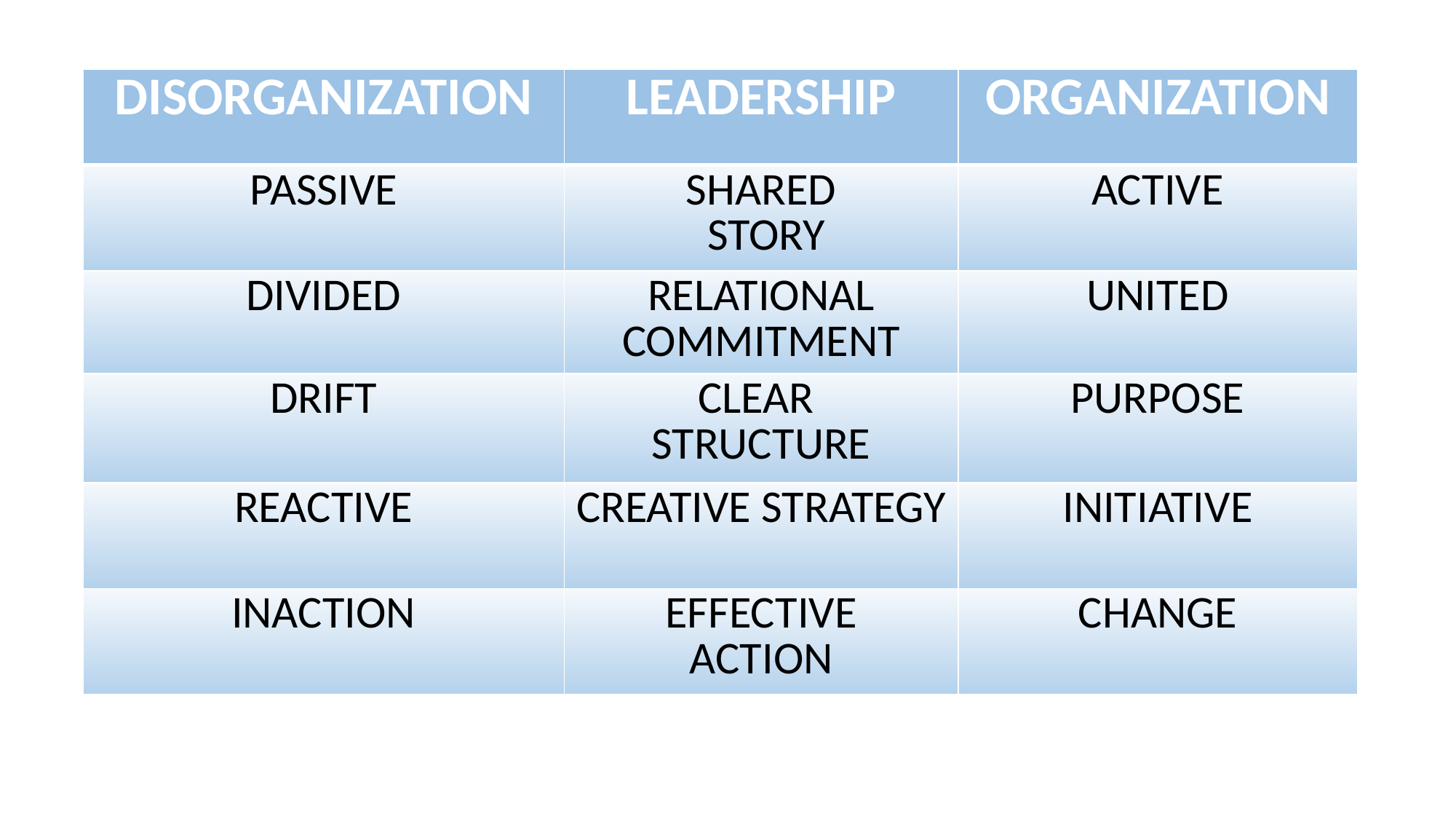

| DISORGANIZATION | LEADERSHIP | ORGANIZATION |
| --- | --- | --- |
| PASSIVE | SHARED STORY | ACTIVE |
| DIVIDED | RELATIONAL COMMITMENT | UNITED |
| DRIFT | CLEAR STRUCTURE | PURPOSE |
| REACTIVE | CREATIVE STRATEGY | INITIATIVE |
| INACTION | EFFECTIVE ACTION | CHANGE |

## Slide 14
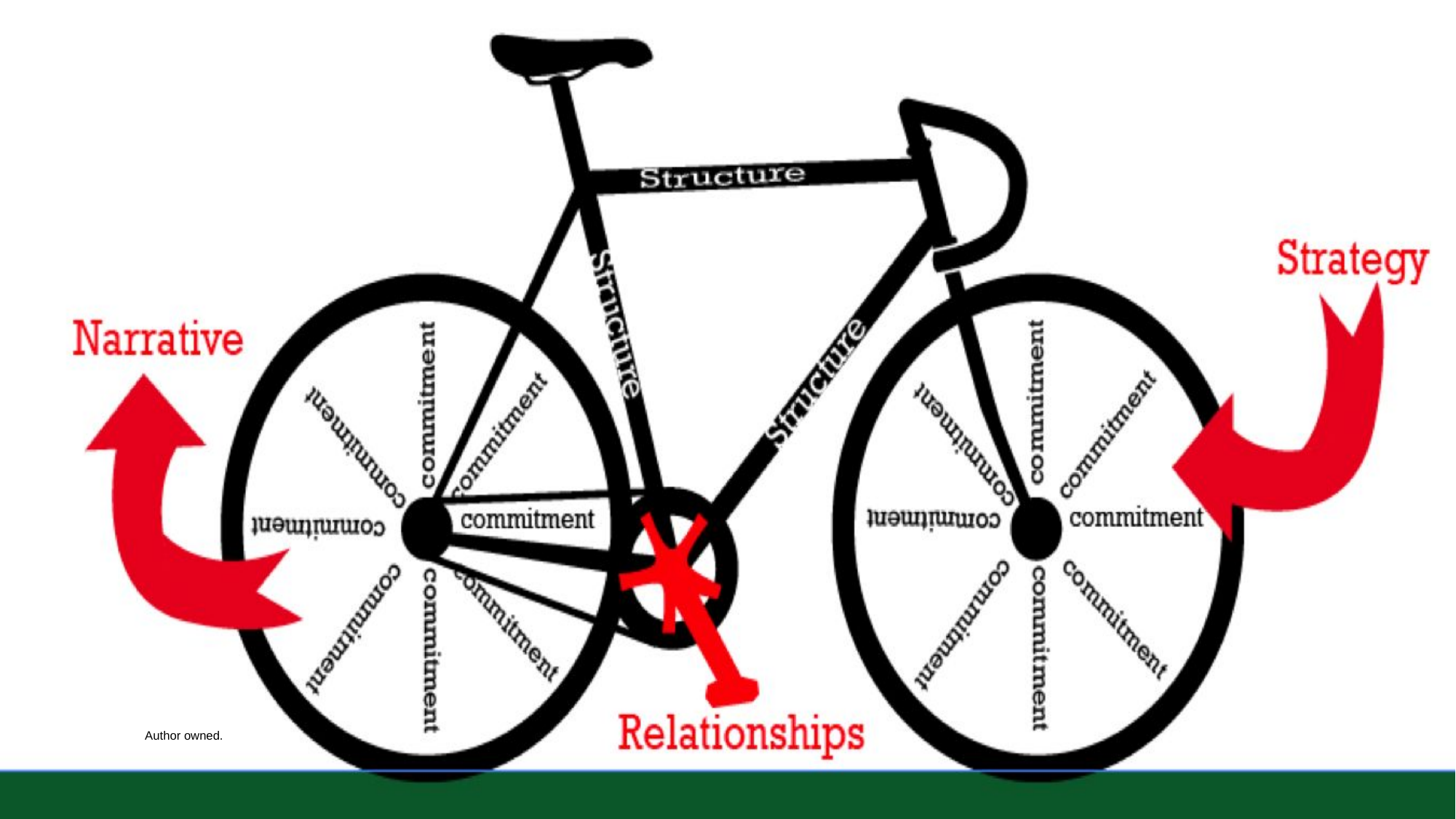

Author owned.
