# Supplementary material for: Preparing Doctors in Training for Health Activist Roles: A Cross-Institutional Community Organizing Workshop for Incoming Medical Residents
Source: MedEdPORTAL. 2022 Jan 18;18:11208. doi: 10.15766/mep_2374-8265.11208 (PMC8763867; doi:10.15766/mep_2374-8265.11208)
Supplement: Supplementary file 1 — Introduction to Community Organizing.pptxIntroduction to Public Narrative.pptxPredrag Stojicic Video.mp4Facilitator Manual.docxStory of Self Small-Group Guide.docxPostworkshop Survey.docx [file mep_2374-8265.11208-s001.zip › B. Introduction to Public Narrative.pptx]

## Slide 1
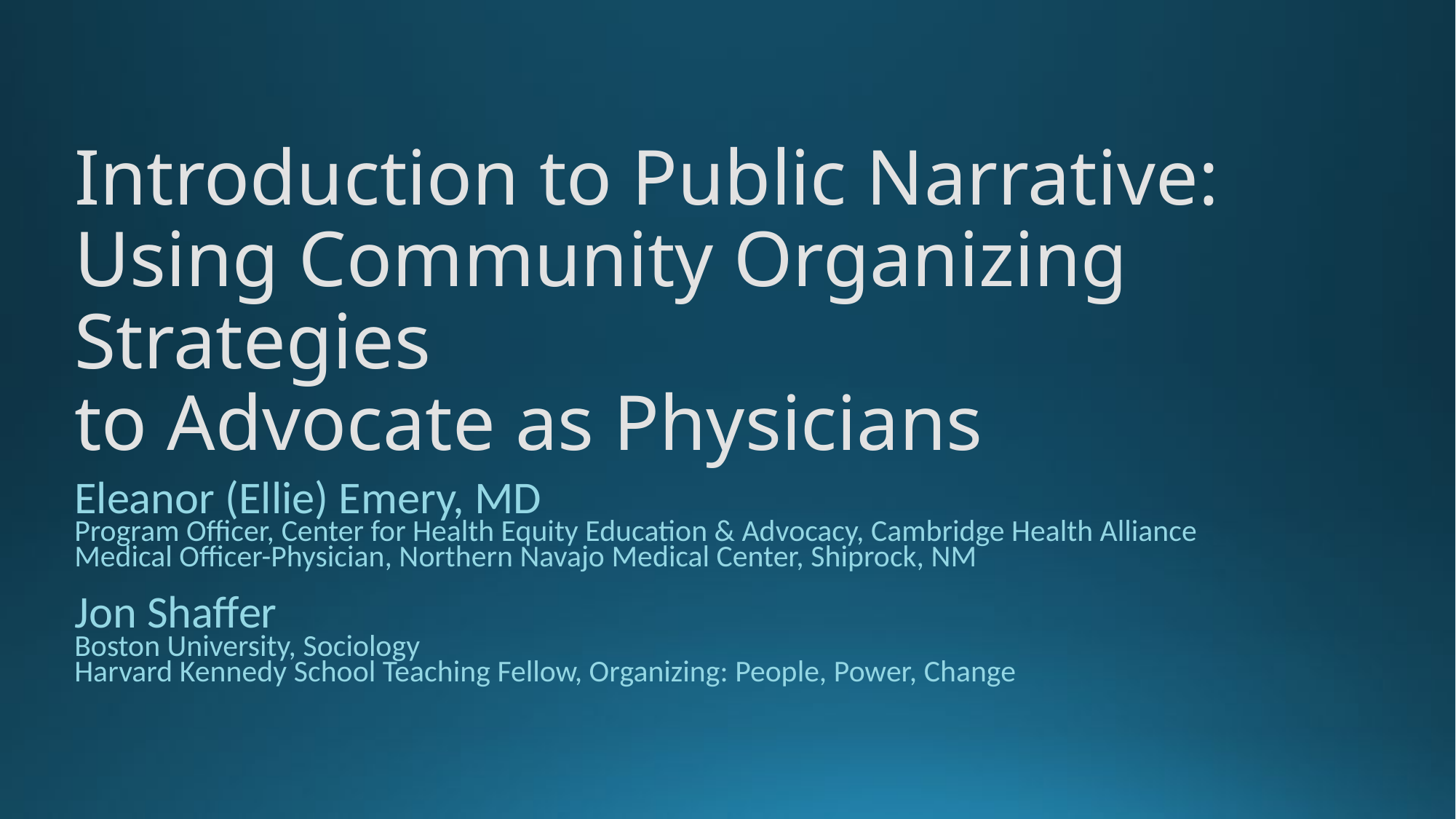

# Introduction to Public Narrative:
Using Community Organizing Strategies
to Advocate as Physicians
Eleanor (Ellie) Emery, MD
Program Officer, Center for Health Equity Education & Advocacy, Cambridge Health Alliance
Medical Officer-Physician, Northern Navajo Medical Center, Shiprock, NM
Jon Shaffer
Boston University, Sociology
Harvard Kennedy School Teaching Fellow, Organizing: People, Power, Change

## Slide 2
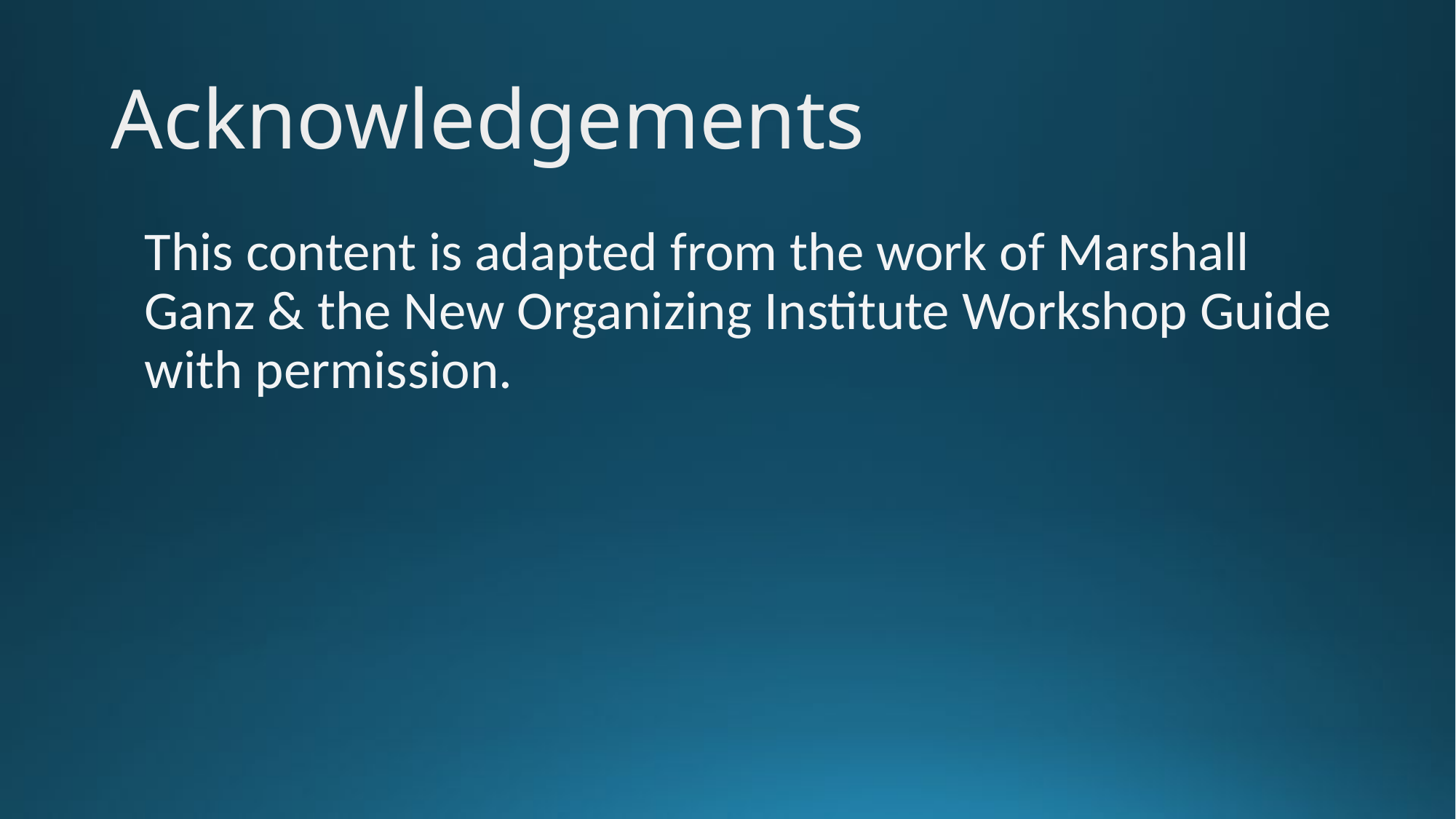

# Acknowledgements
This content is adapted from the work of Marshall Ganz & the New Organizing Institute Workshop Guide with permission.

## Slide 3
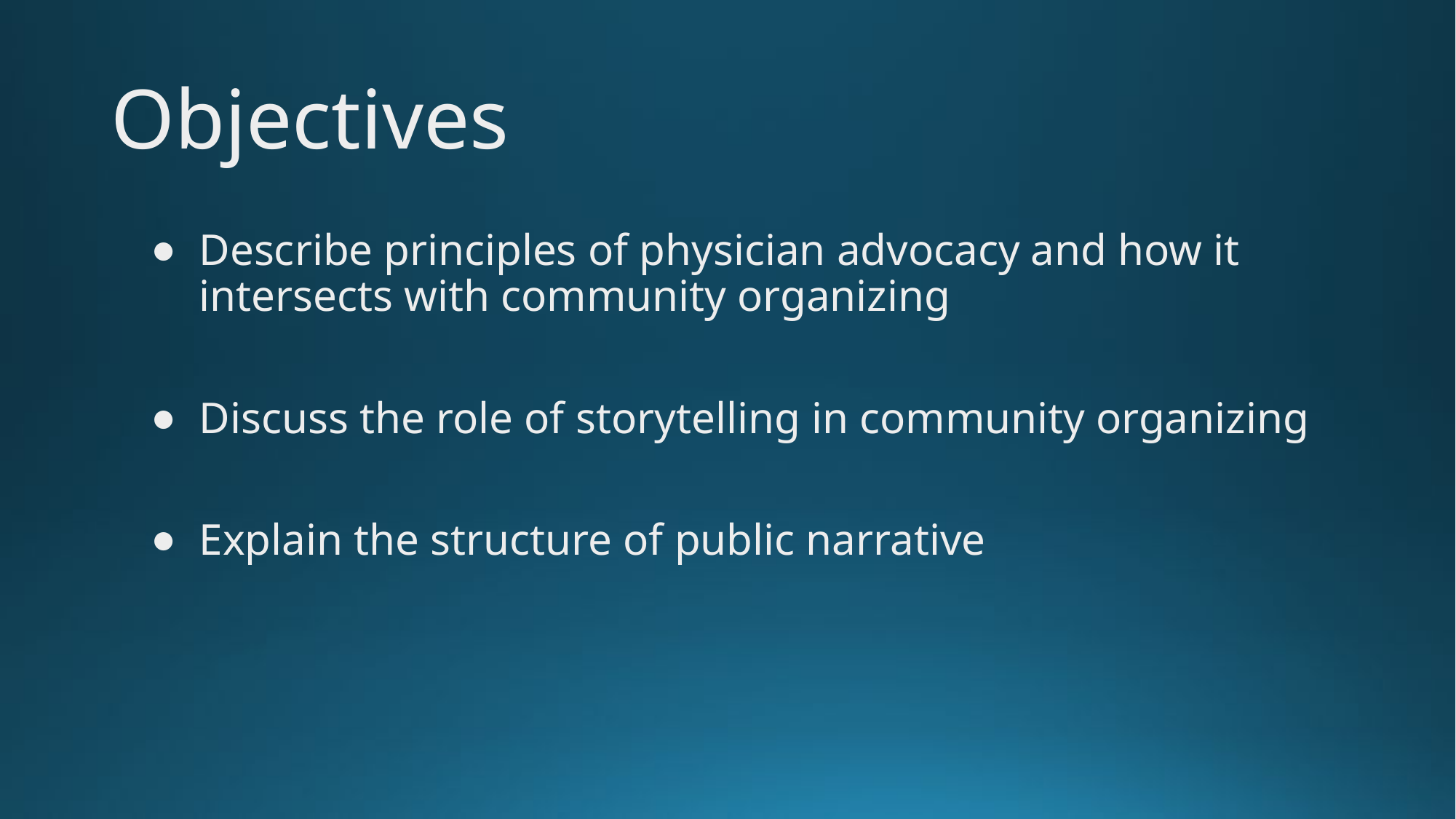

# Objectives
Describe principles of physician advocacy and how it intersects with community organizing
Discuss the role of storytelling in community organizing
Explain the structure of public narrative

## Slide 4
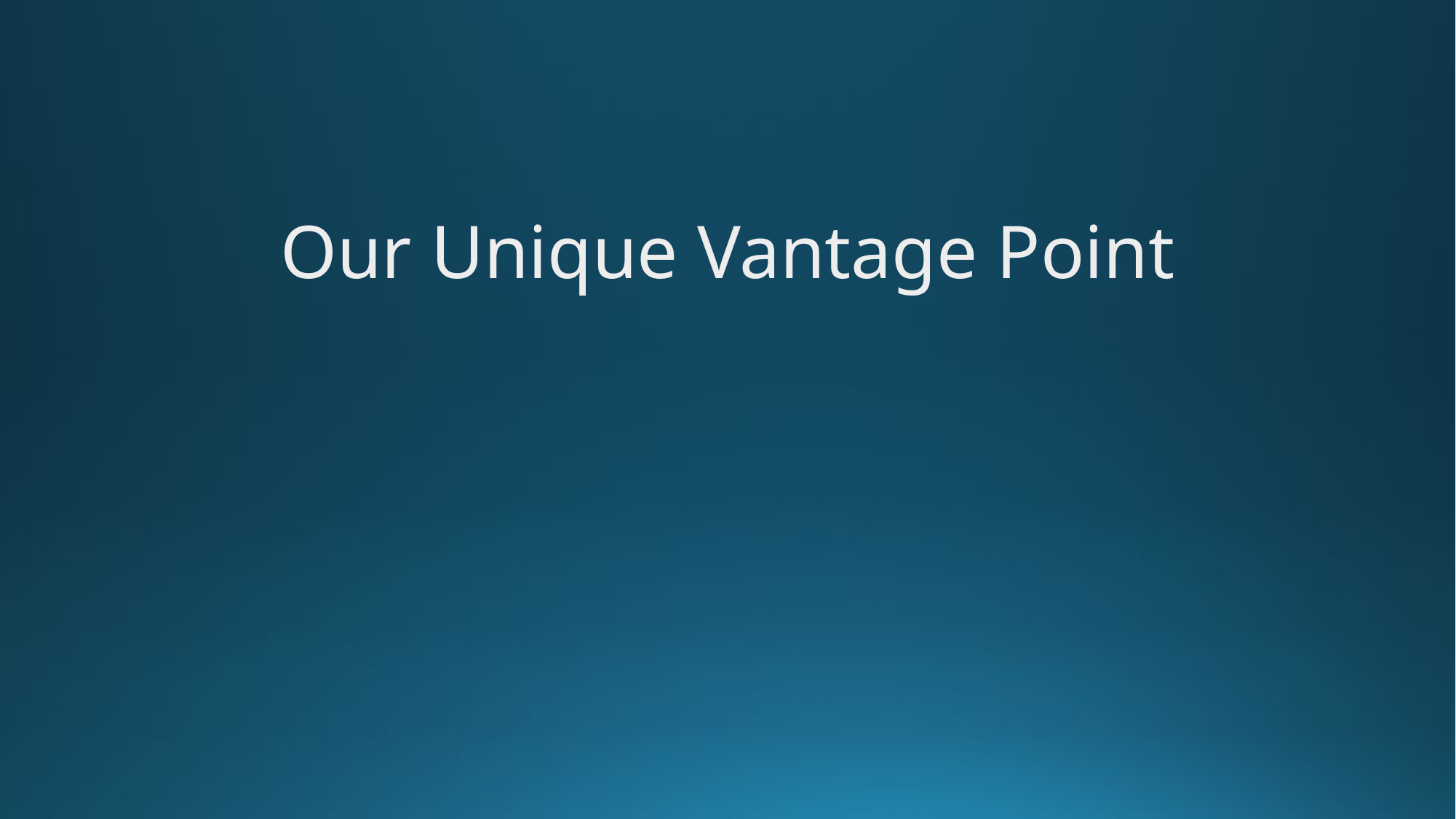

# Our Unique Vantage Point

## Slide 5
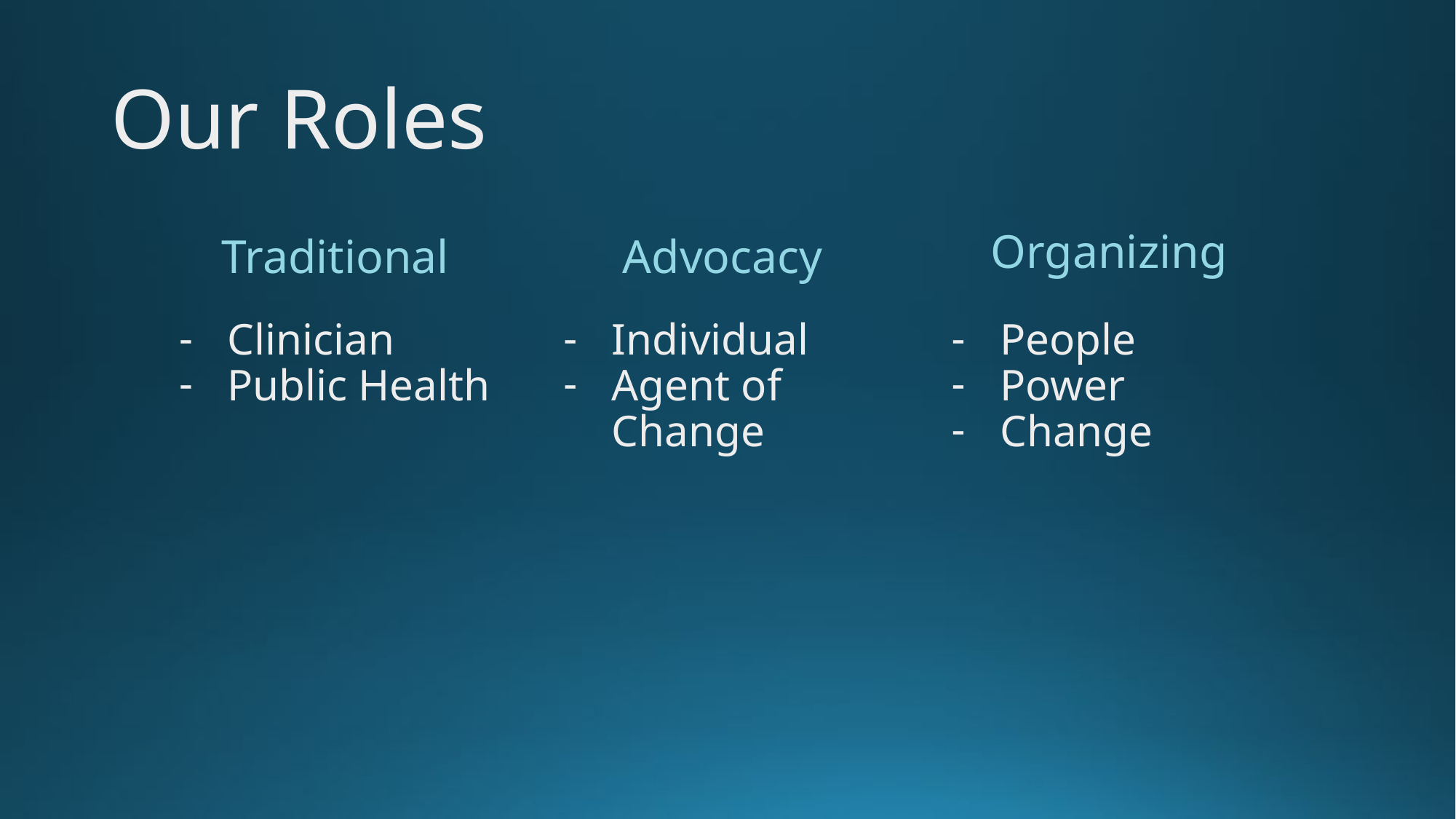

# Our Roles
Organizing
Traditional
Advocacy
Clinician
Public Health
Individual
Agent of Change
People
Power
Change

## Slide 6
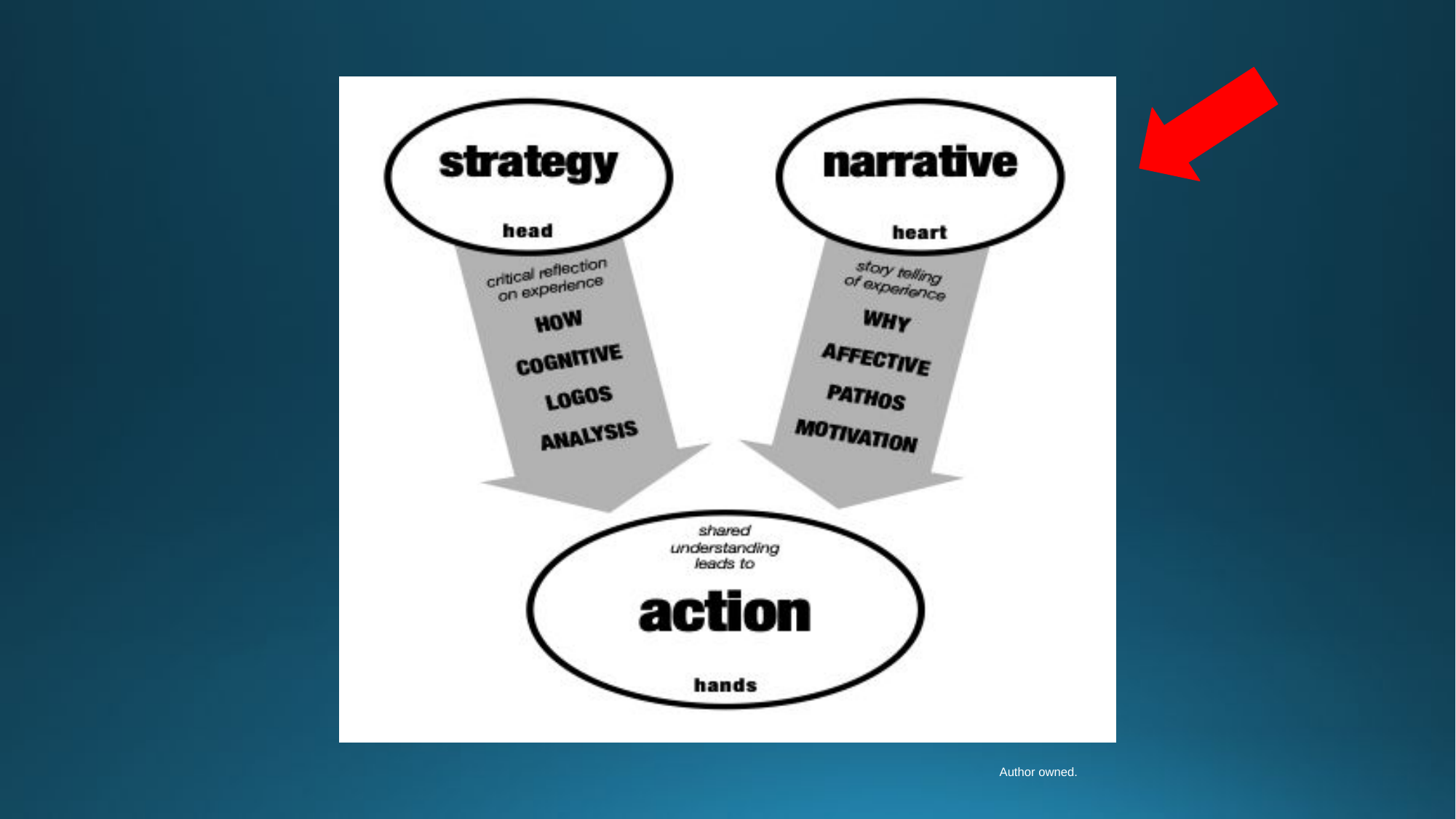

Author owned.

## Slide 7
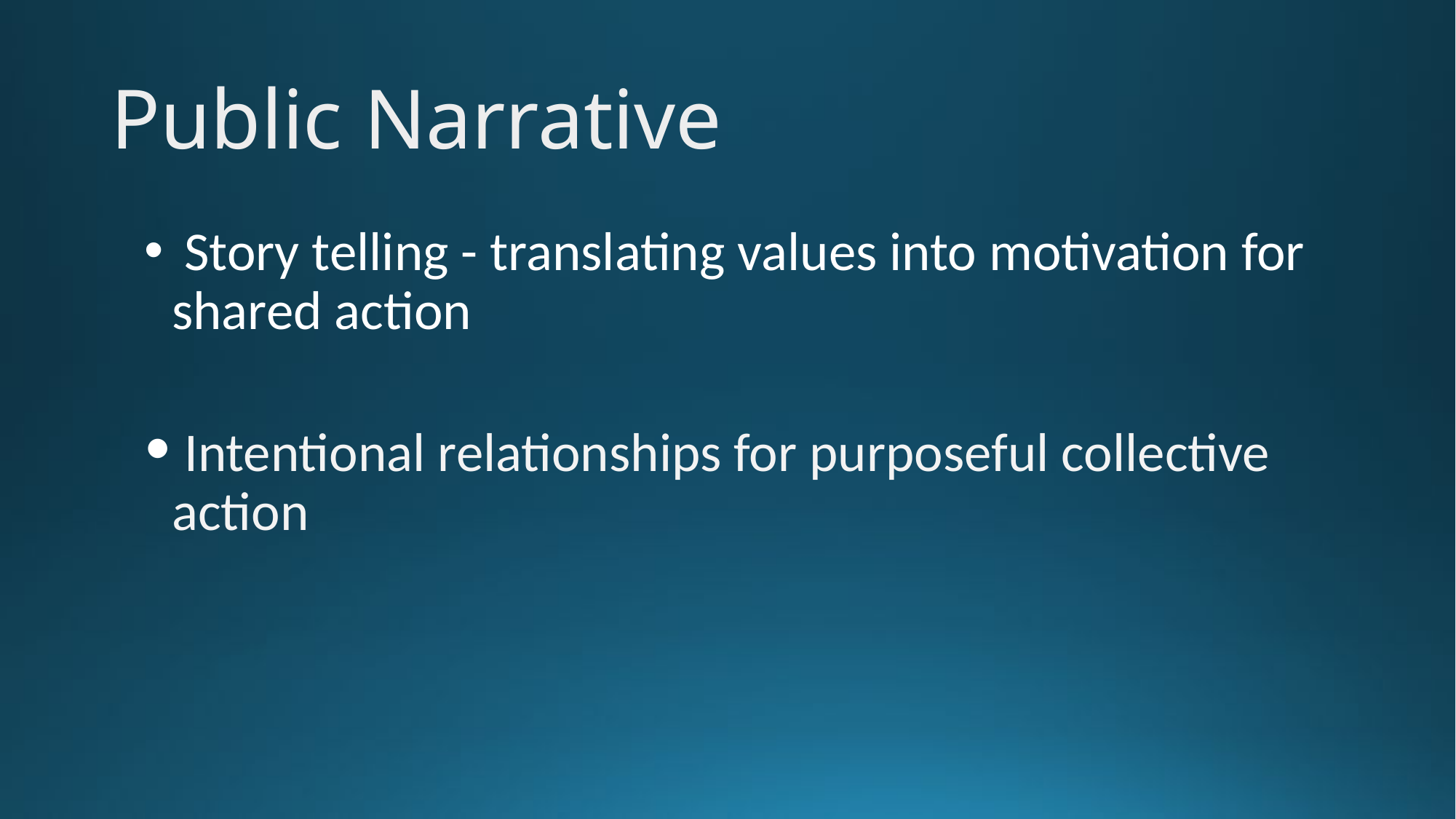

# Public Narrative
 Story telling - translating values into motivation for shared action
 Intentional relationships for purposeful collective action

## Slide 8
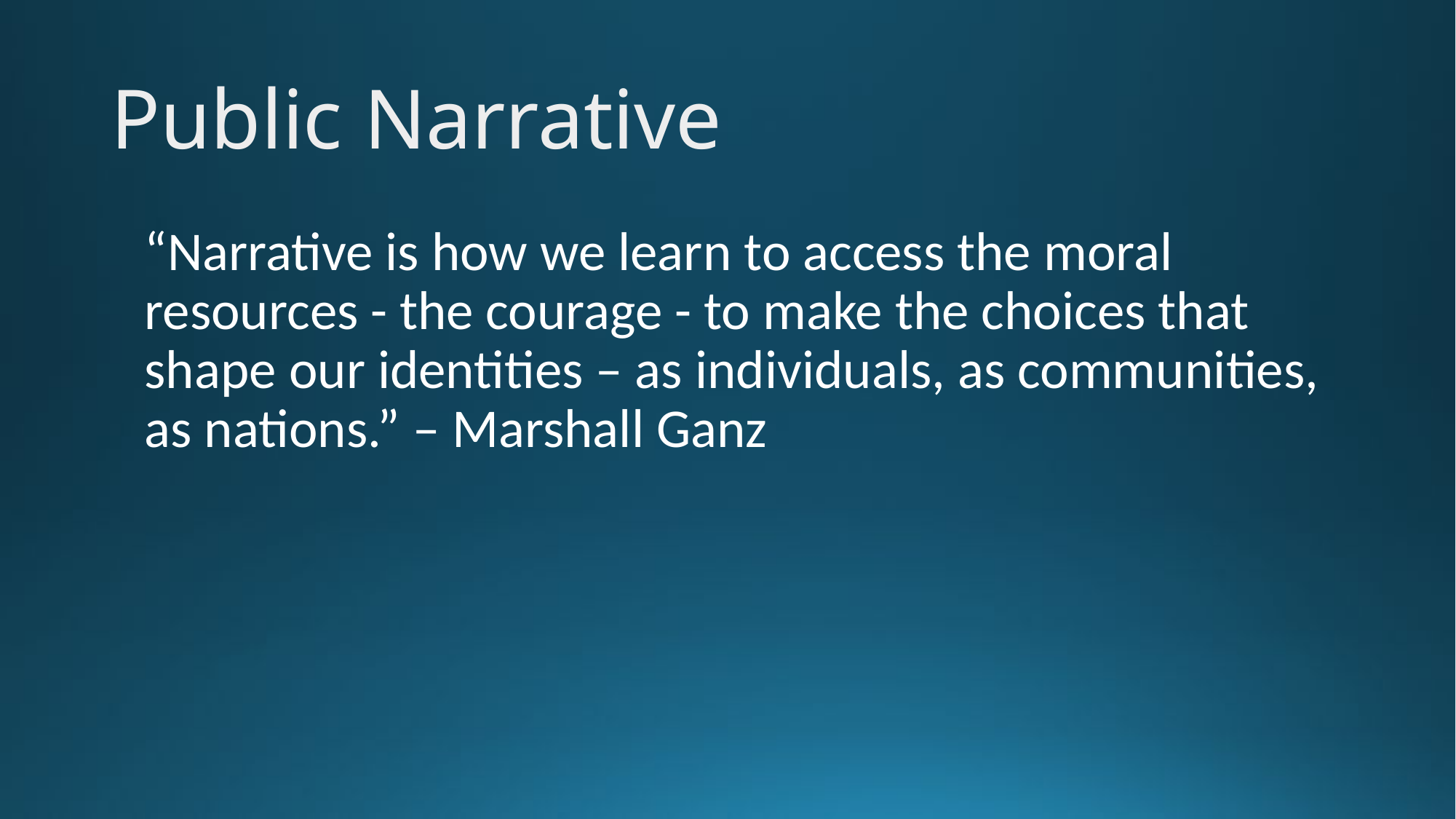

# Public Narrative
“Narrative is how we learn to access the moral resources - the courage - to make the choices that shape our identities – as individuals, as communities, as nations.” – Marshall Ganz

## Slide 9
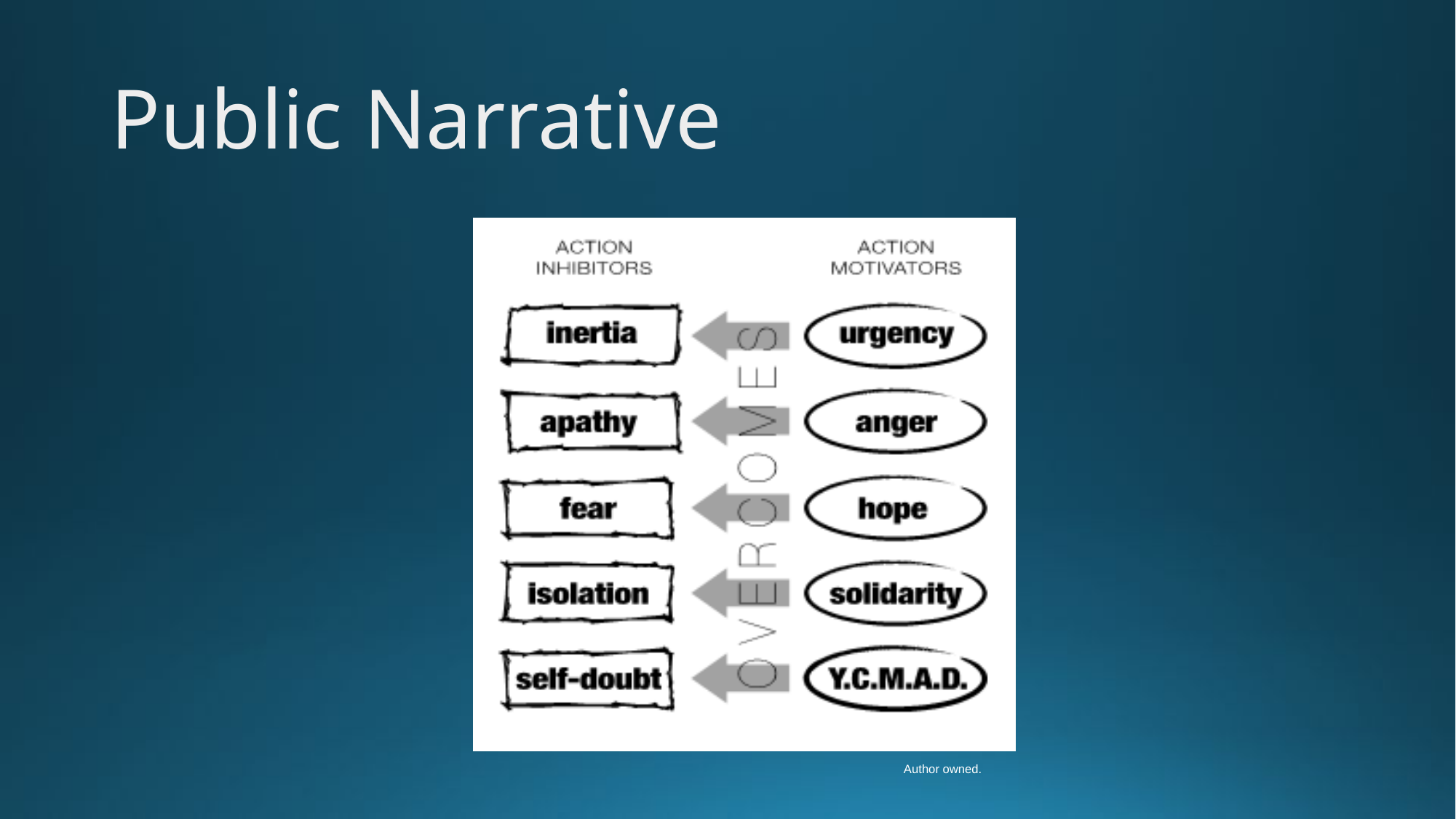

# Public Narrative
Author owned.

## Slide 10
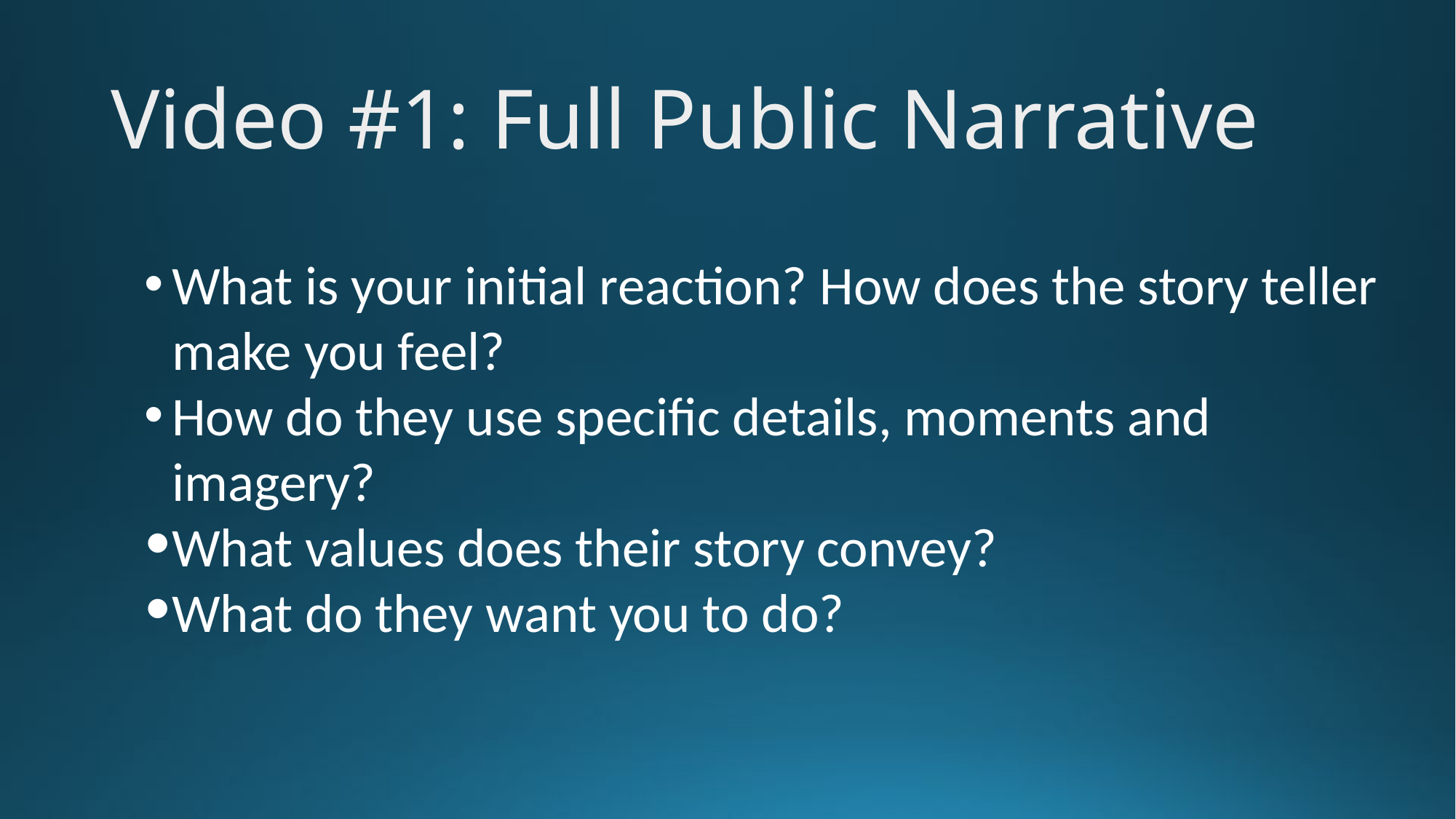

# Video #1: Full Public Narrative
What is your initial reaction? How does the story teller make you feel?
How do they use specific details, moments and imagery?
What values does their story convey?
What do they want you to do?

## Slide 11
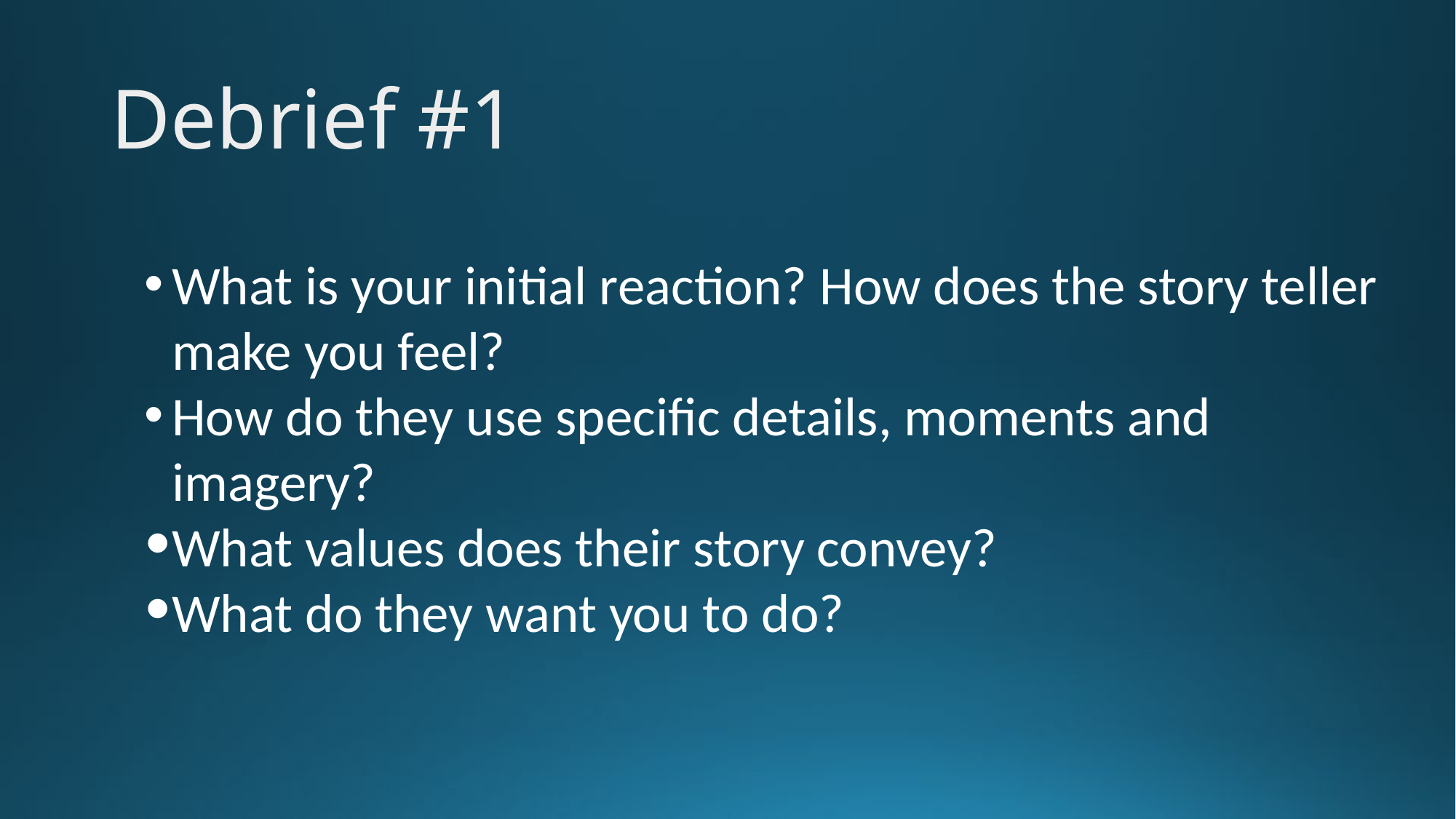

# Debrief #1
What is your initial reaction? How does the story teller make you feel?
How do they use specific details, moments and imagery?
What values does their story convey?
What do they want you to do?

## Slide 12
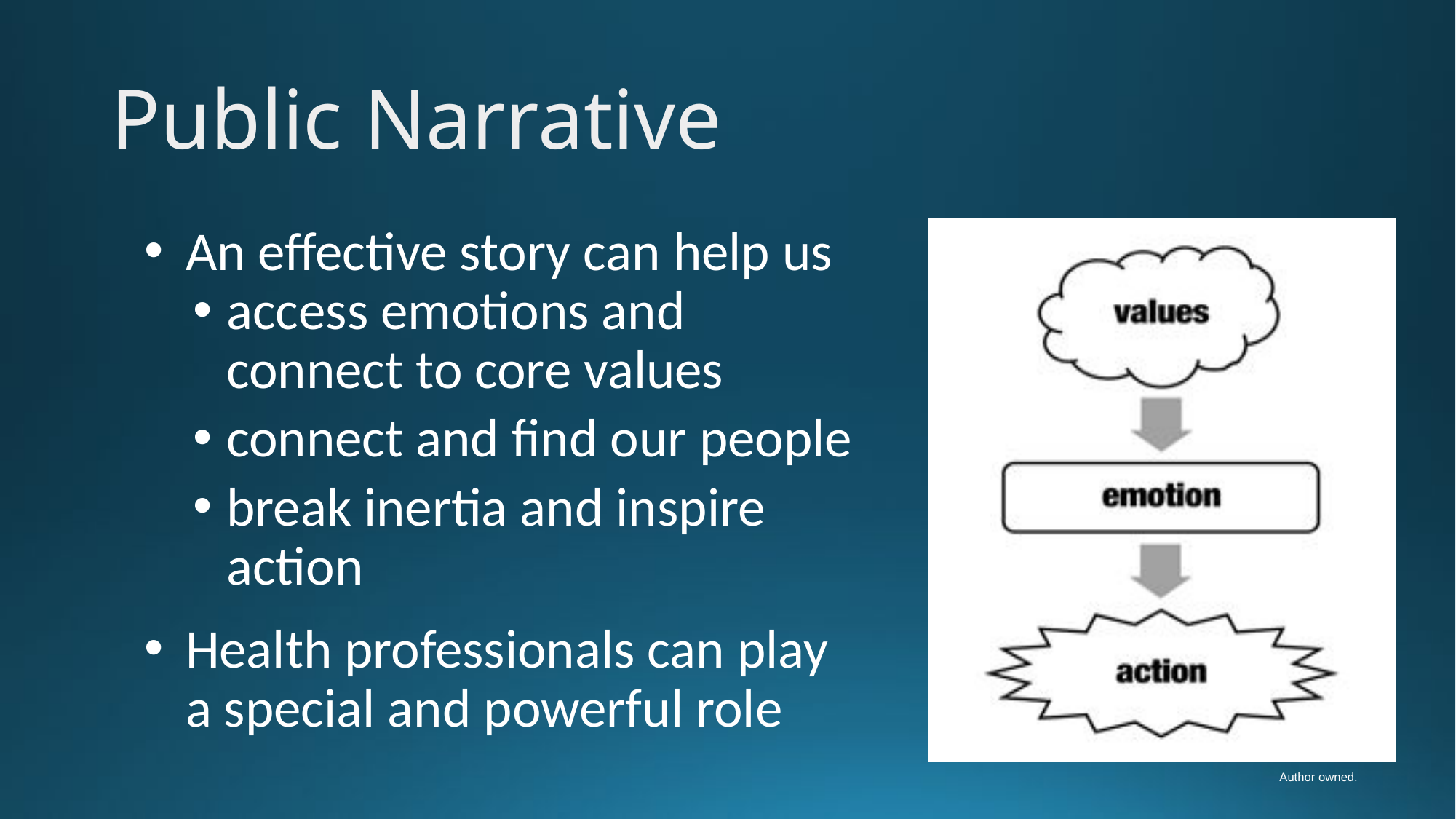

# Public Narrative
An effective story can help us
access emotions and connect to core values
connect and find our people
break inertia and inspire action
Health professionals can play a special and powerful role
Author owned.

## Slide 13
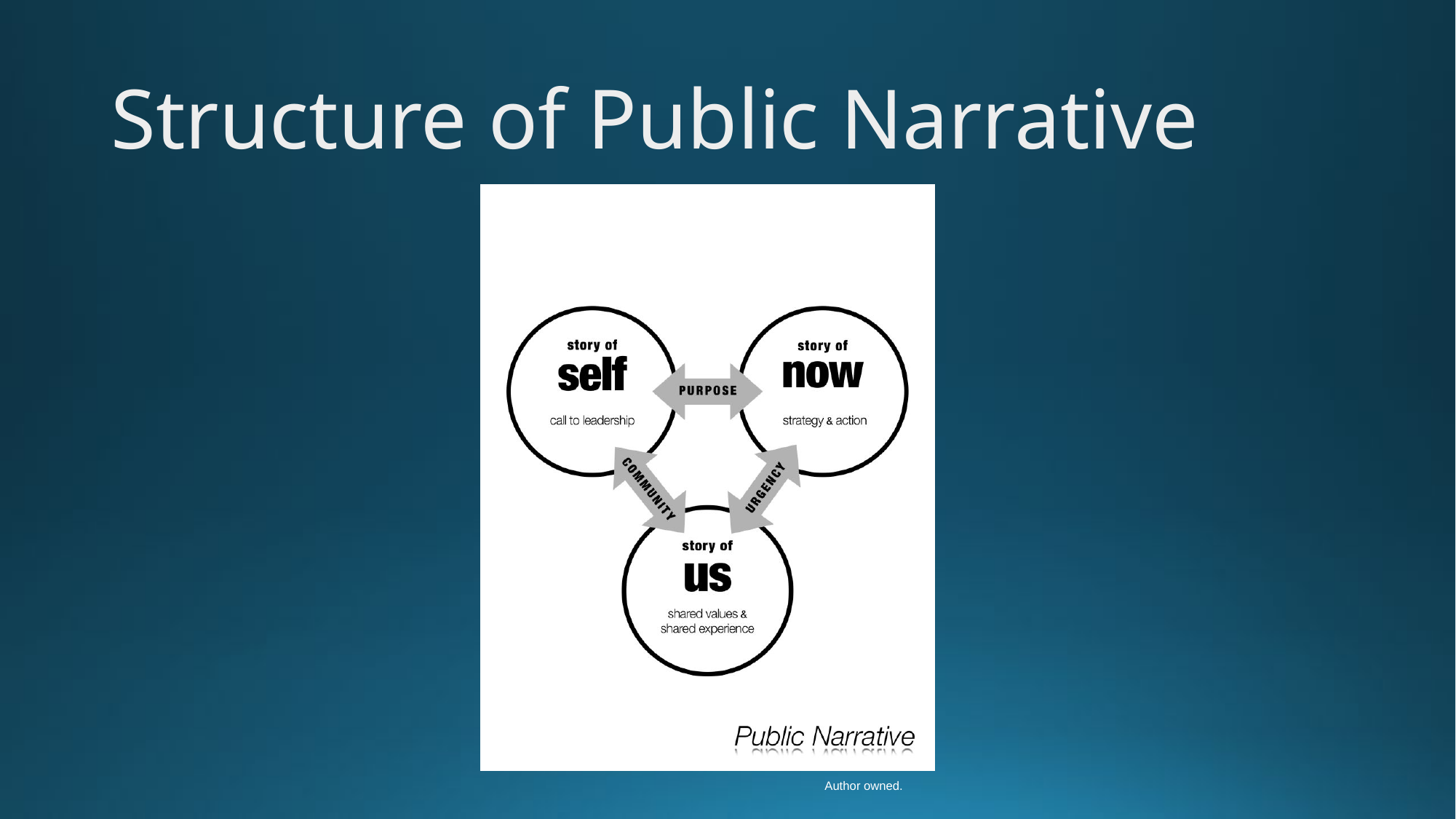

# Structure of Public Narrative
Author owned.

## Slide 14
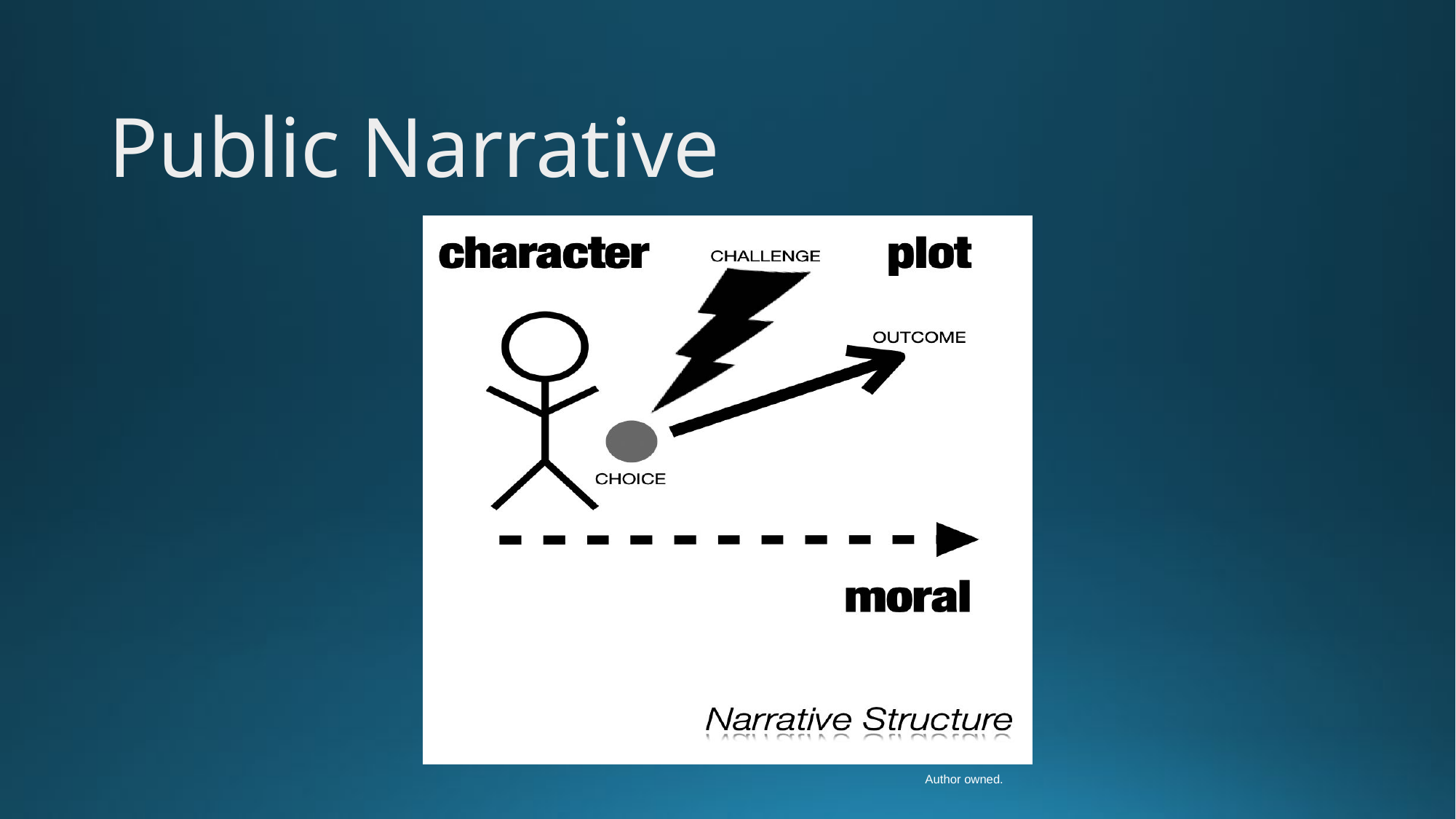

Public Narrative
Author owned.

## Slide 15
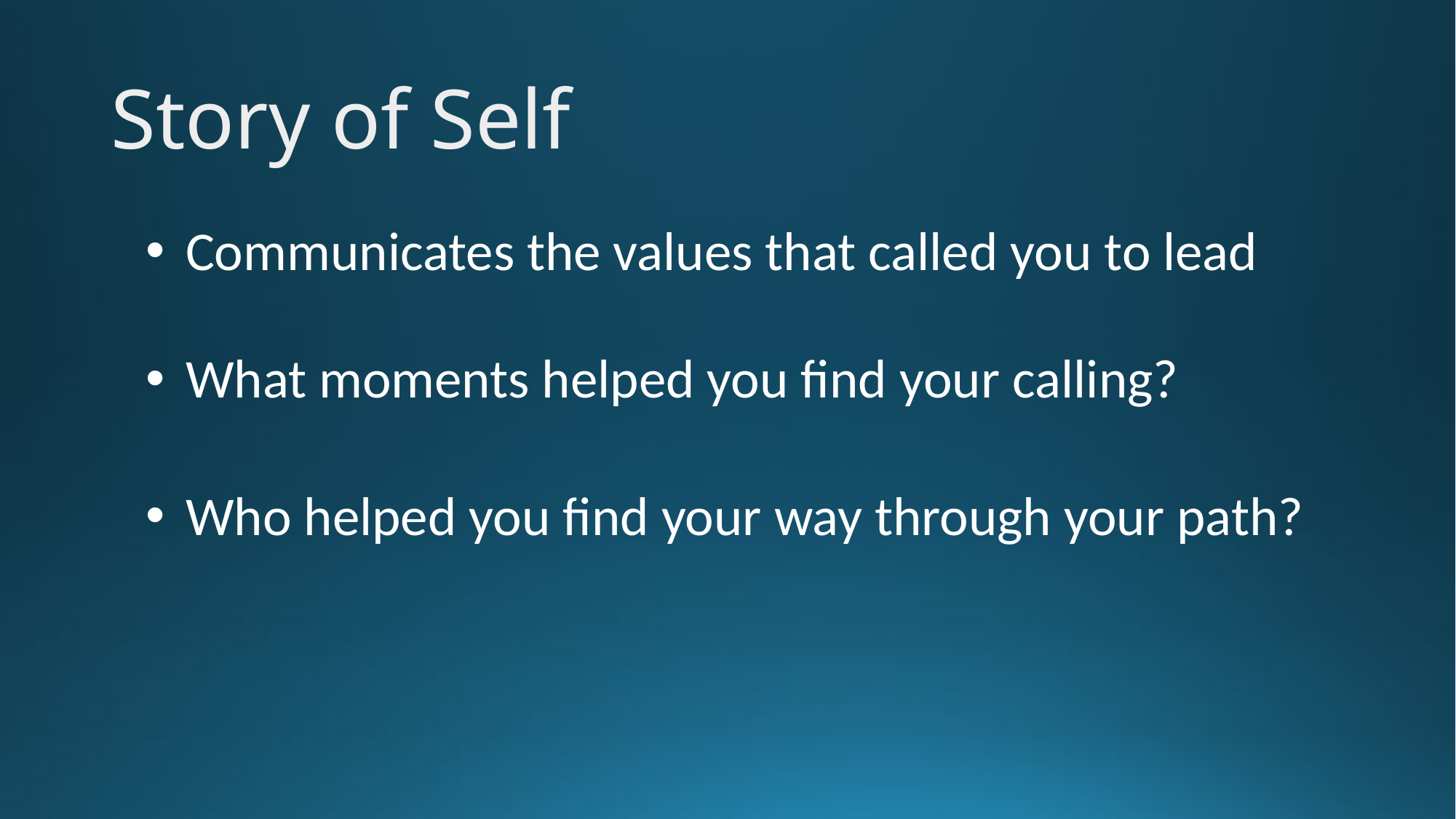

# Story of Self
Communicates the values that called you to lead
What moments helped you find your calling?
Who helped you find your way through your path?

## Slide 16
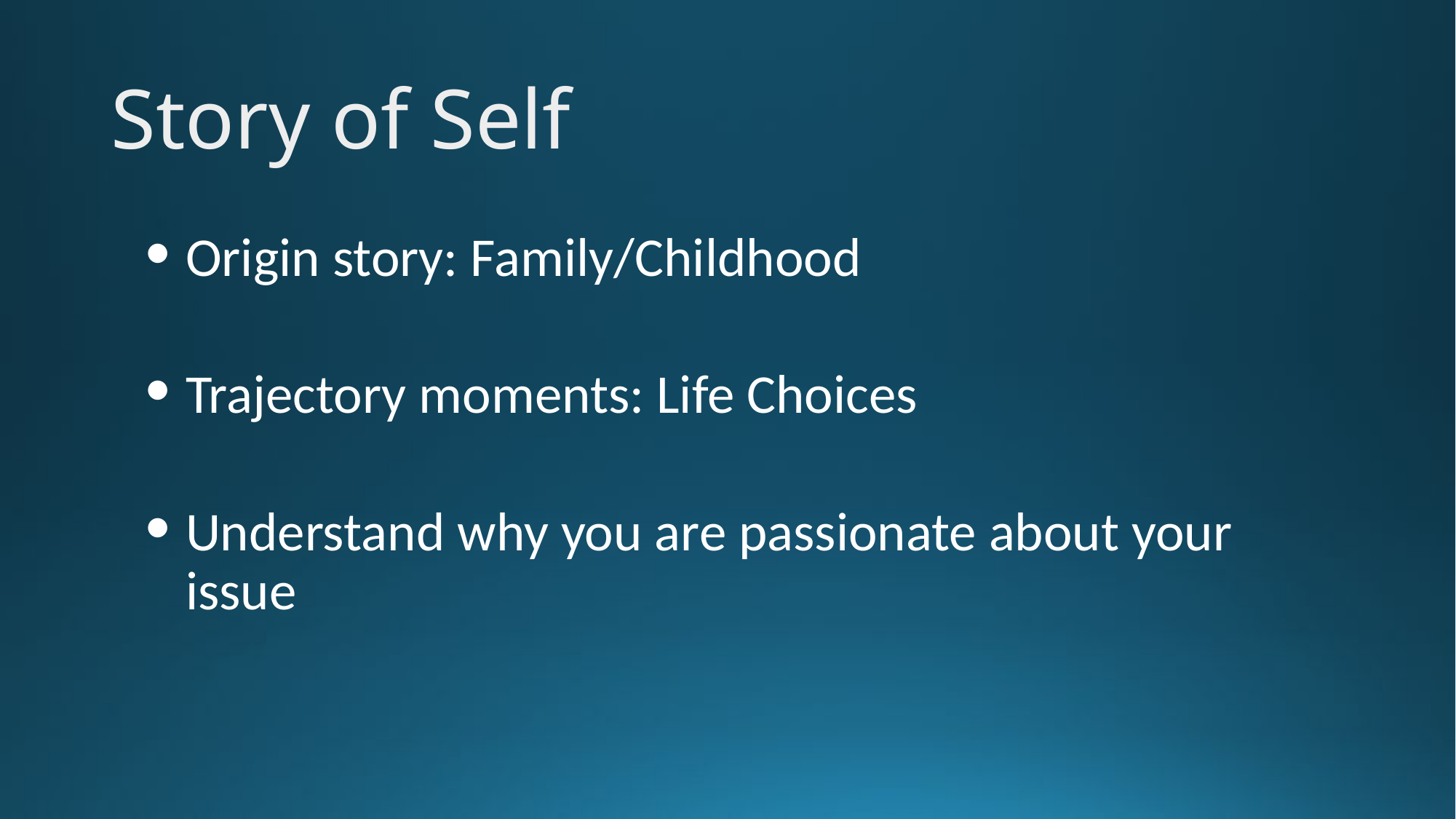

# Story of Self
Origin story: Family/Childhood
Trajectory moments: Life Choices
Understand why you are passionate about your issue

## Slide 17
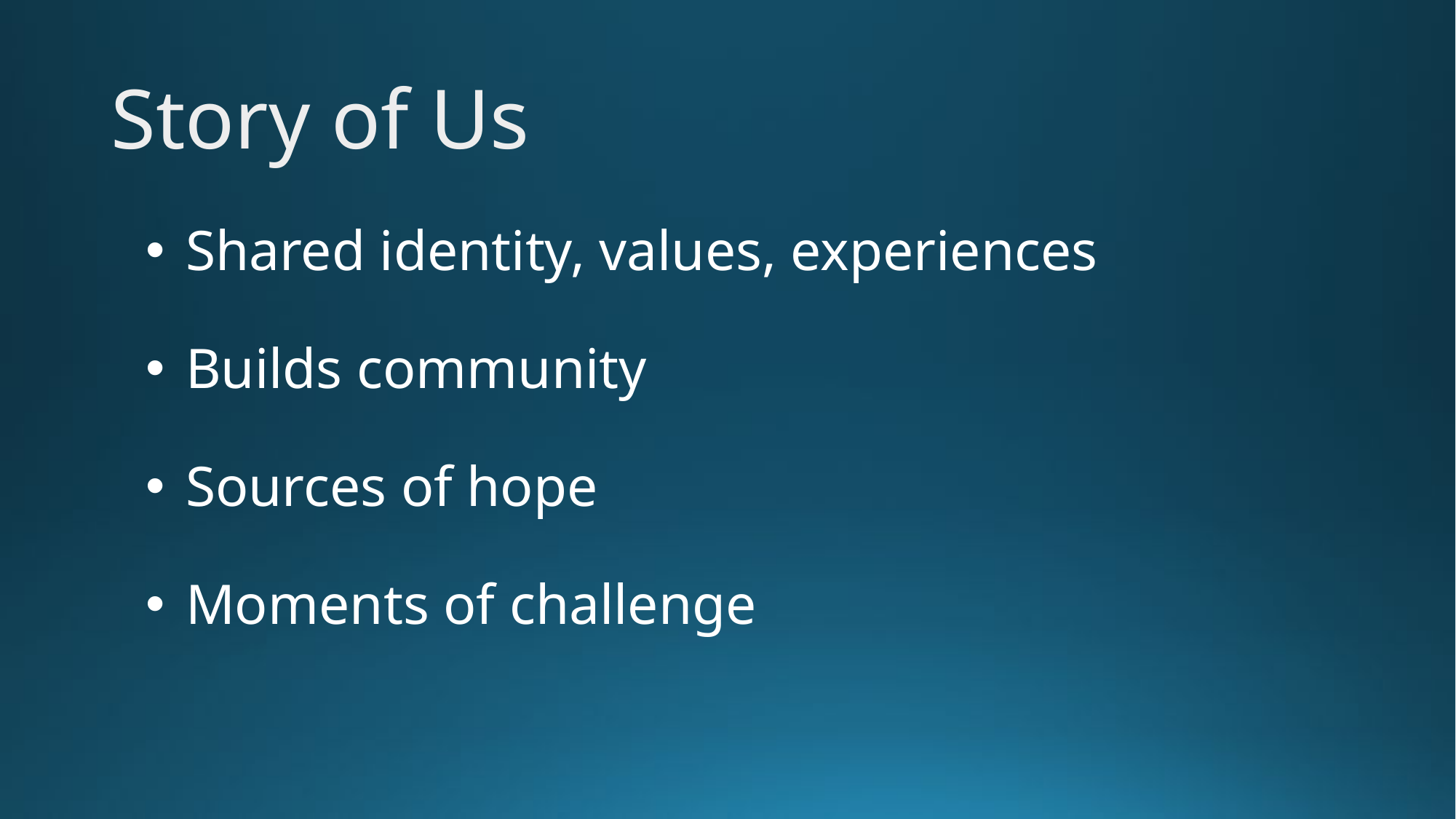

# Story of Us
Shared identity, values, experiences
Builds community
Sources of hope
Moments of challenge

## Slide 18
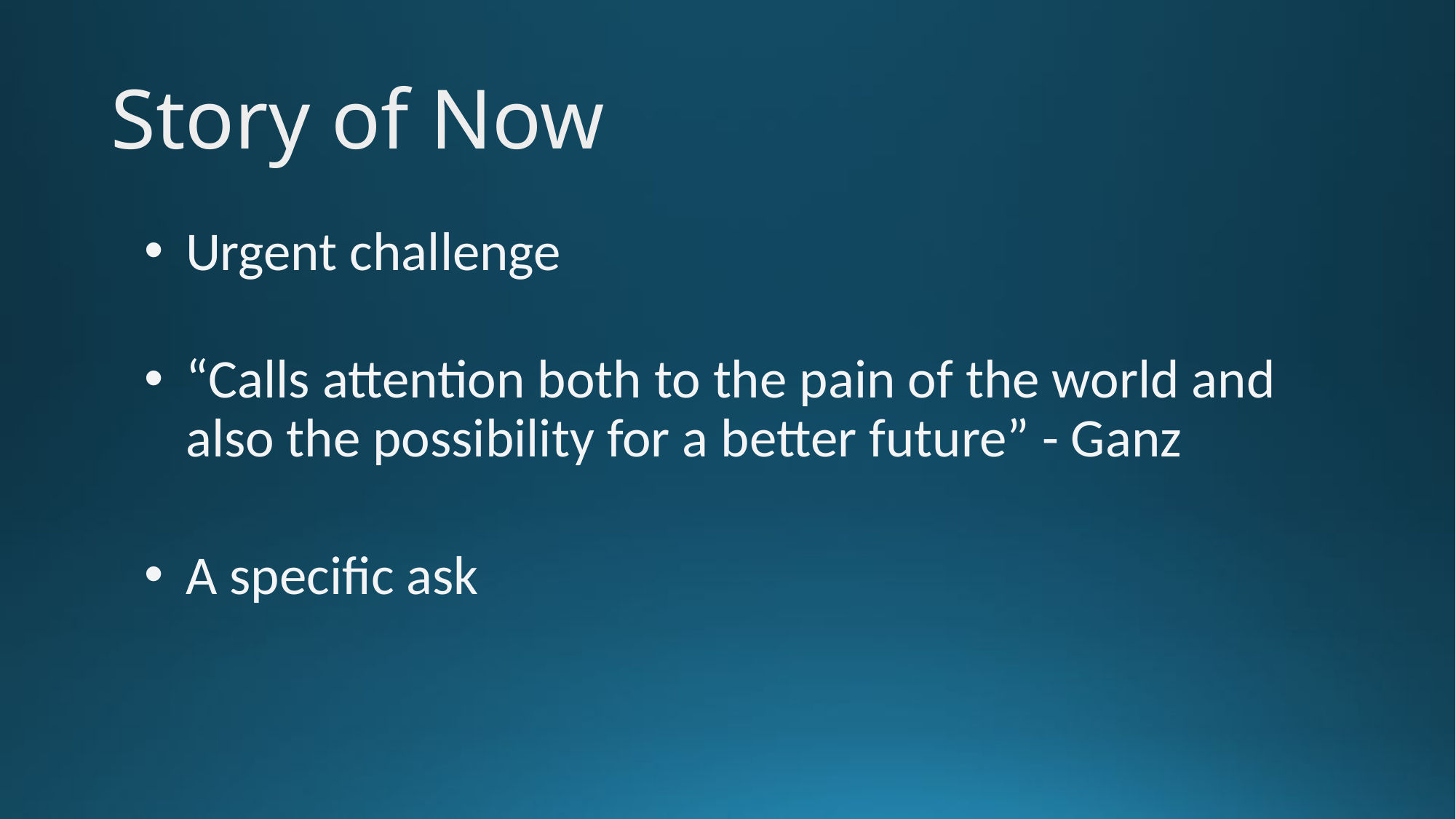

# Story of Now
Urgent challenge
“Calls attention both to the pain of the world and also the possibility for a better future” - Ganz
A specific ask

## Slide 19
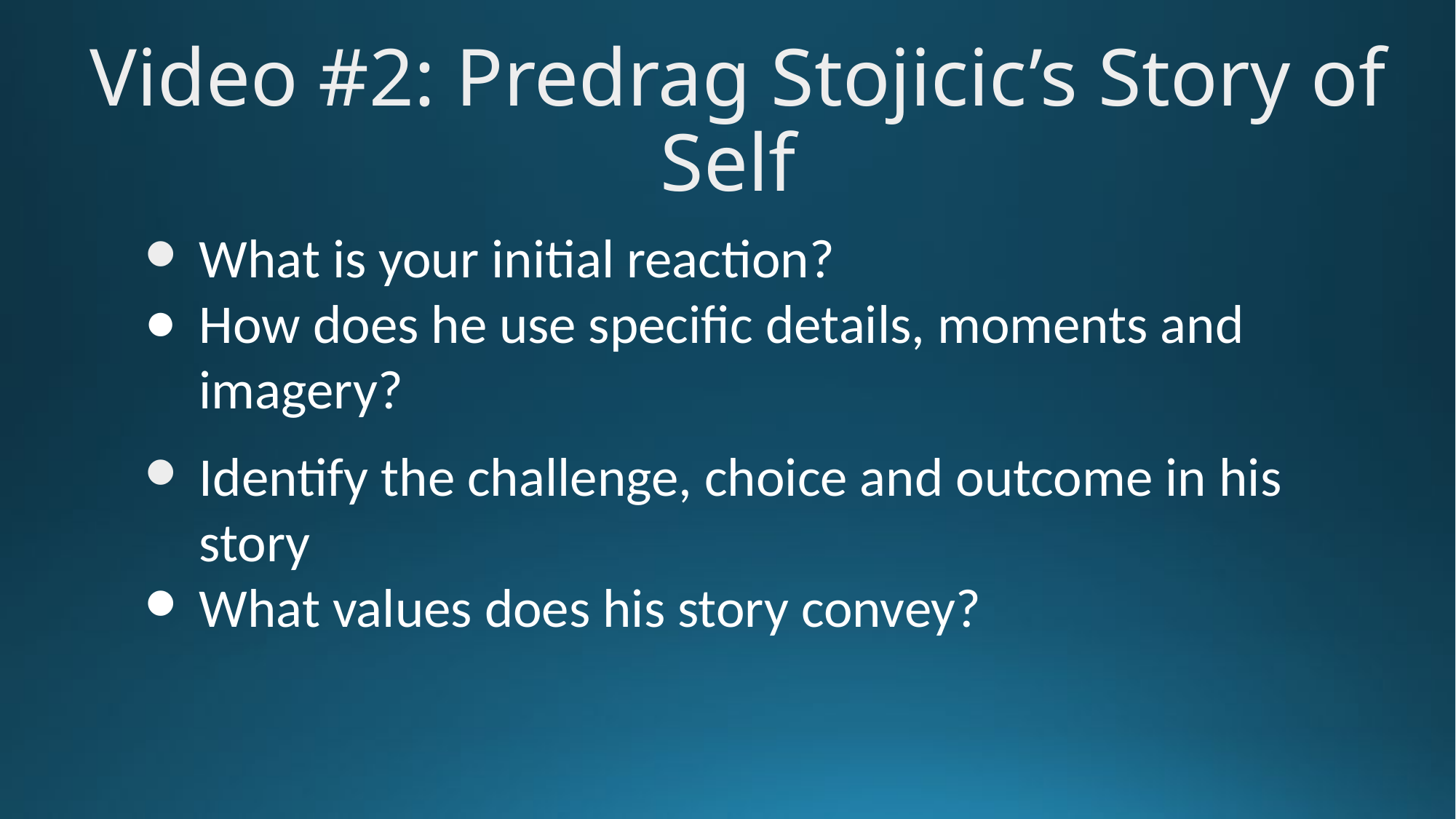

# Video #2: Predrag Stojicic’s Story of Self
What is your initial reaction?
How does he use specific details, moments and imagery?
Identify the challenge, choice and outcome in his story
What values does his story convey?

## Slide 20
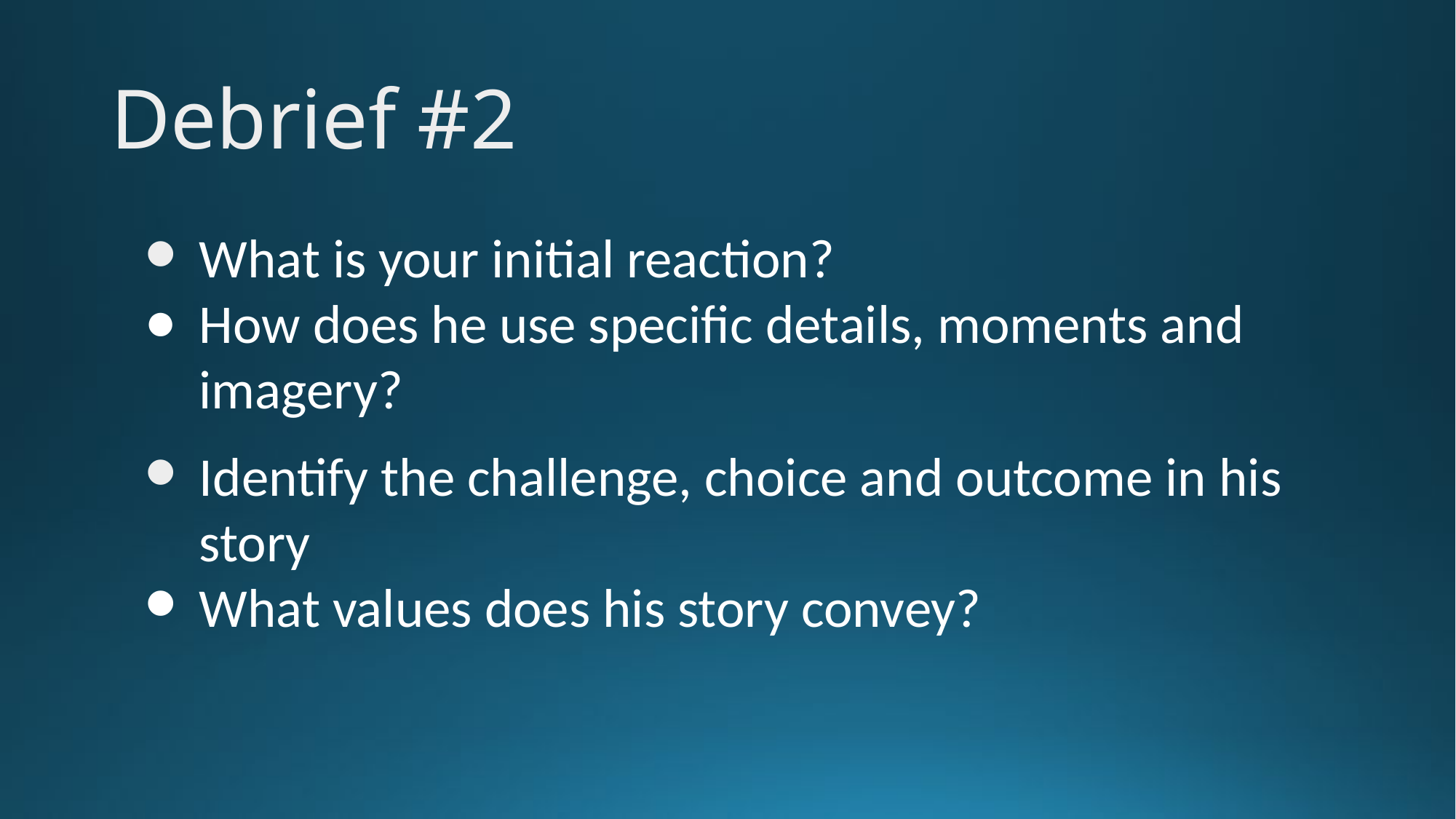

# Debrief #2
What is your initial reaction?
How does he use specific details, moments and imagery?
Identify the challenge, choice and outcome in his story
What values does his story convey?

## Slide 21
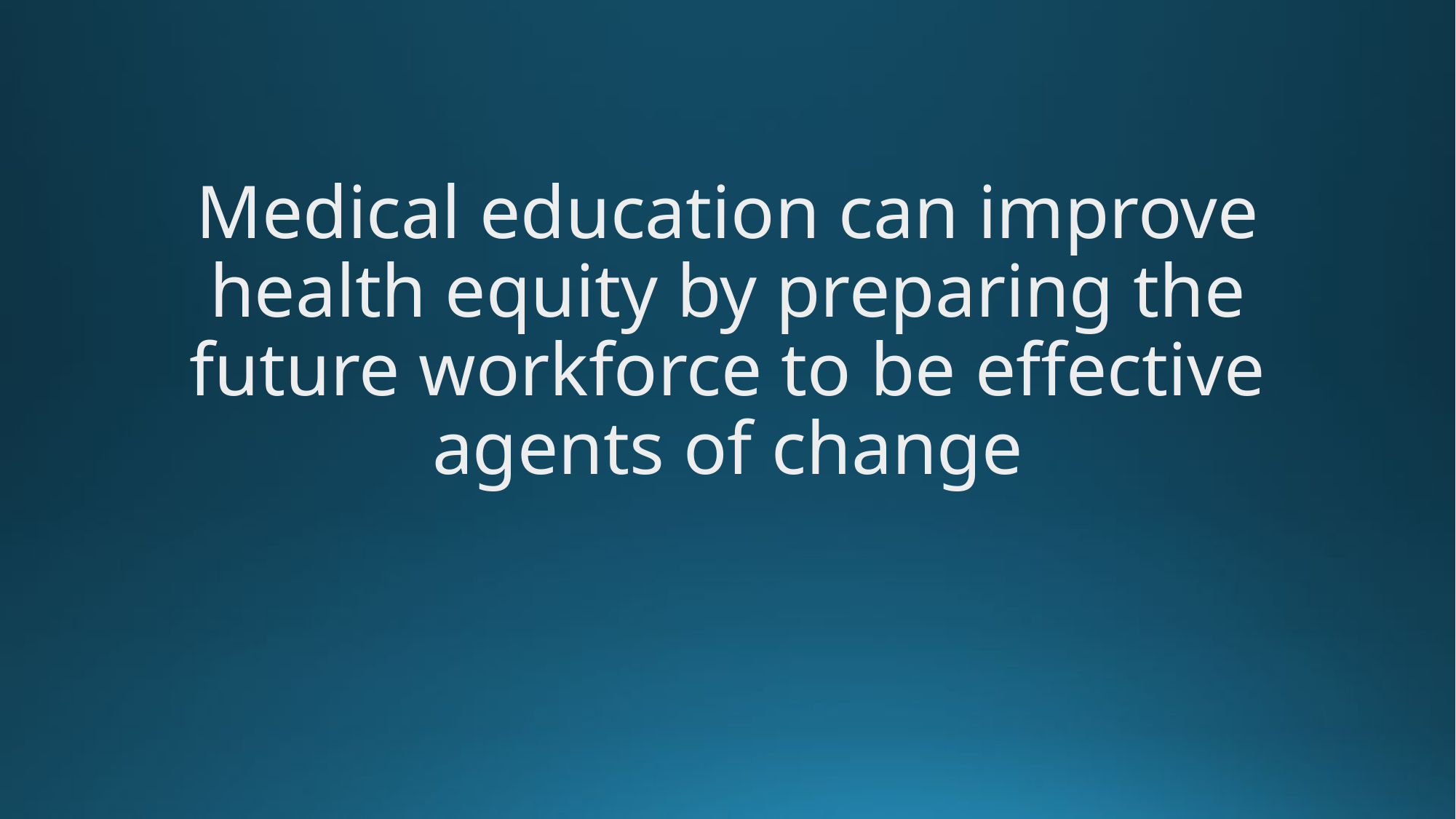

# Medical education can improve health equity by preparing the future workforce to be effective agents of change
