# Supplementary material for: Preparing Doctors in Training for Health Activist Roles: A Cross-Institutional Community Organizing Workshop for Incoming Medical Residents
Source: MedEdPORTAL. 2022 Jan 18;18:11208. doi: 10.15766/mep_2374-8265.11208 (PMC8763867; doi:10.15766/mep_2374-8265.11208)
Supplement: Supplementary file 1 — Introduction to Community Organizing.pptxIntroduction to Public Narrative.pptxPredrag Stojicic Video.mp4Facilitator Manual.docxStory of Self Small-Group Guide.docxPostworkshop Survey.docx [file mep_2374-8265.11208-s001.zip › D. Facilitator Manual.docx]

**Story of Self Workshop**

**Facilitator Guide**

### *June 8th, 2019*

CAMBRIDGE, MA

Developed for this training by Jonathan Shaffer, Eleanor Emery and Gaurab Basu

**Overview**

The purpose of this facilitator guide is to support educators interested in delivering a public narrative story of self workshop. Prior to facilitating this event, we recommend that educators leading this workshop complete an online or in-person community organizing training and coach at least one public narrative workshop, such as those offered through the Leading Change Network (see Suggested Resources below). In this guide we have included instructions on how to introduce the different components of the workshop and move between them. Speaker notes for the PowerPoints (Appendices A and B) are included directly in the PowerPoints.

**Educational Objectives**By the end of this activity, learners will be able to:

1. Identify the key concepts of community organizing.

2. Identify the key concepts of one specific community organizing leadership skill, public narrative.

3. Apply the principles of public narrative by developing their own story of self.

4. Present a story of self to other learners in the session.

5. Describe one way in which public narrative skills could be utilized to support health equity work during residency training.

**Workshop Handouts and Materials**

1. Story of Self Small Group Guide (Appendix E)
   1. This can be handed out during the break that precedes the Introduction to Story of Self Small groups
2. Post-Workshop Survey (Appendix F)

**Suggested Resources**

1. Full public narrative video example: Croft J. 6.12 Seconds – James Croft’s Harvard LGBT Bullying Speech [Video]. YouTube. <https://www.youtube.com/watch?v=lymvc5d6qxY>. Published November 8, 2012. Accessed September 12, 2021.
   1. This publicly available video can be used as a model of a full public narrative during the Introduction to Public Narrative.
2. The Leading Change Network: <https://leadingchangenetwork.org/>
   1. This organization offers regular trainings in public narrative and community organizing. Facilitators and coaches can participate in these trainings to hone their skills in public narrative and community organizing as well as in coaching prior to leading this workshop. The website also features a “Learning Resources” page with many useful resources on both public narrative and community organizing.

**Suggested Agenda and Timeline**

| **Time** | **Agenda** |
| --- | --- |
| 15 minutes | Welcome & Introductions |
| 20 minutes | Introduction to Community Organizing |
| 30 minutes | Introduction to Public Narrative |
| 10 minutes | Break |
| 10 minutes | Introduction to Story of Self Small Groups |
| 45 minutes | Story of Self Small Groups |
| 20 minutes | Large Group Story of Self Sharing Session |
| 20 minutes | Large Group Discussion |
| 10 minutes | Close & Complete Evaluations |

**Welcome & Introductions**

Open the session with a few words about the importance of health equity and being equipped to make systemic change to improve the health of our patients. One effective way to do this is to have one faculty leader introduce themself by delivering a public narrative focused on what calls them this work and why they believe it is relevant for this group and at this moment in time (story of self, us and now). The premise of this workshop is that physicians can benefit from developing leadership skills to augment their clinical skills. In this case, the workshop seeks to develop an awareness of community organizing leadership skillsets and provide an opportunity to workshop one specific skill called public narrative.

Following this introduction, the other leaders and/or coaches involved in the session should briefly introduce themselves, their professional background, and what led them to participate in this community organizing workshop.

The leader should then introduce the educational objectives for the workshop and review the agenda for the day (see above). The leader should then solicit a set of norms that the group collectively agrees to abide by for the duration of the workshop, such as be on time (return for any breaks promptly), confidentiality (no personal stories that are discussed should be shared outside the group without permission) and “step up, step back” (if you normally speak often, try listening, and if you are normally more reserved try speaking up). One of the co-leaders can write the norms that the group suggests on a whiteboard so that they remain visible throughout the workshop. The leader should also have the group identify a “norm correction,” or something that the group members agree to do if they violate a norm. This is often something quick and silly, such as patting your head while rubbing your stomach. The goal is not to embarrass or punish someone for violating a norm, but rather to acknowledge a deviation from the agreed upon norms, because not doing so would render them meaningless.

**Introduction to Community Organizing**See Appendix A for PowerPoint with detailed speaker notes.

**Introduction to Public Narrative**

See Appendix B for PowerPoint with detailed speaker notes. The story of self video example used in this presentation is provided in Appendix C.

**Introduction to Story of Self Small Groups**

The following is suggested wording for introducing the story of self small groups:

*We have just reviewed public narrative, one of the five organizing leadership practices. We will now be splitting up into small groups so that you can all have the opportunity to practice telling the first piece of your own narrative, a story of self. While understanding the principles of public narrative and story of self are important, this skill cannot be learned just by reading slides and seeing examples; it must be practiced. Developing a skillful story in the short allotment of time we have today will be challenging. The point is not to have a polished story of self, but rather to give it a try and get a sense of whether this narrative practice could be valuable to you in both distilling the values that motivate you to lead and communicating these values to others. We hope this introduction will also pique your interest in getting additional training to further develop this skill.*

*A story of self workshop guide is found in Appendix E. Page 3 of this guide shows an agenda for the small groups. After we split up, everyone will get five minutes to brainstorm their story of self. Page 4 of Appendix E includes a worksheet that will help you brainstorm for your story of self using the principles just discussed. Each participant will then have two minutes to take a stab at telling their story. Afterwards your small group coach will take three minutes to coach you on your story while also soliciting feedback from your other group members. Page 5-6 of Appendix E includes tips and a worksheet to assist you in coaching your other small groupmates.*

To demonstrate how this works, have one leader or coach who has been identified in advance (ideally a different person from the one who shared their public narrative in the Introduction) share their 2 minute story of self in front of the full group . Then have a second leader or coach take 3 minutes to coach them just as we will do in the small groups. After this exercise is complete, split the group up into the small groups that you have assigned prior to the start of the session, ideally with a ratio one coach to five participants.

**Story of Self Small Groups**

Each coach will lead their small group using the agenda outlined on page 3 of Appendix E.

At the end of this exercise before returning to the large group, the coach of each small group should identify one individual who told a powerful story of self and privately ask them if they would be interested in re-telling their story to the larger group. The coach can encourage them by highlighting the strengths of the story they shared but should also give them permission to decline if they do not feel comfortable. If they decline, the leader can ask a different individual.

**Large Group Sharing Session & Discussion**

After the story of self small groups, all participants come back together in the larger group.

As above, the coach of each small group will have sought to identity one participant from their group who would be interested in retelling their story of self to the larger group. Having participants present to the larger group seeks to accomplish a few goals:

1. It generates a sense of communal support for and celebration of the story teller and the bravery they show by presenting their story of self to the large group.
2. It allows participants to have more exposure to peers who demonstrate effectiveness in this skillset.
3. It ends the workshop with a sense of camaraderie and communal pride on a job well done.

Each participant is given 2 minutes to present their story of self, and there is no coaching or peer feedback, only cheers and applause.

After each of the pre-selected individuals have shared, if time allows, the leader can then ask if anyone else would like to share their story.

Once the sharing is complete, the session closes with a debrief. The workshop leaders ask participants to share “pluses,” things that went well or ways they found the workshop effective, and “deltas,” or things they would change or improve. To facilitate this discussion, the leader can re-present the educational objectives included at the beginning of this guide. The leaders should also ask participants how they imagined themselves employing this skill in their work and get a sense of what next step goals and interests the group has, including whether they are interested in participating in a full public narrative workshop (story of self, us and now) and/or workshops on other community organizing leadership skills.

**Close & Complete Evaluations**

Finally, the leader can close by naming something they found powerful or moving about the session and thanking the participants for their engagement.

All participants should then be asked to fill out the evaluation forms before leaving for the day.
