# Supplementary material for: Preparing Doctors in Training for Health Activist Roles: A Cross-Institutional Community Organizing Workshop for Incoming Medical Residents
Source: MedEdPORTAL. 2022 Jan 18;18:11208. doi: 10.15766/mep_2374-8265.11208 (PMC8763867; doi:10.15766/mep_2374-8265.11208)
Supplement: Supplementary file 1 — Introduction to Community Organizing.pptxIntroduction to Public Narrative.pptxPredrag Stojicic Video.mp4Facilitator Manual.docxStory of Self Small-Group Guide.docxPostworkshop Survey.docx [file mep_2374-8265.11208-s001.zip › F. Postworkshop Survey.docx]

**Appendix F.** Post-Workshop Survey Administered to All Participants

Please rate the degree to which you agree or disagree with each of the following statements on a scale from 1 to 5 where 1 is strongly disagree and 5 is strongly agree.

| I am glad I attended this event. | 1 | 2 | 3 | 4 | 5 |
| --- | --- | --- | --- | --- | --- |
| I would participate in a similar event in the future. | 1 | 2 | 3 | 4 | 5 |
| This event helped me feel connected to my motivations for pursuing medicine | 1 | 2 | 3 | 4 | 5 |
| This event helped me learn how to become a more effective physician advocate. | 1 | 2 | 3 | 4 | 5 |
| I am more familiar with the concepts of community organizing (including public narrative, story of self) than I was prior to participating in this event. | 1 | 2 | 3 | 4 | 5 |
| I think the community organizing skillset (including public narrative, story of self) is an important one for a physician to possess. | 1 | 2 | 3 | 4 | 5 |
| I met trainees and faculty members at this event that I may want to collaborate with on future projects. | 1 | 2 | 3 | 4 | 5 |

Please provide any general impressions or feedback you have on this event.
