# Supplementary material for: Preparing Doctors in Training for Health Activist Roles: A Cross-Institutional Community Organizing Workshop for Incoming Medical Residents
Source: MedEdPORTAL. 2022 Jan 18;18:11208. doi: 10.15766/mep_2374-8265.11208 (PMC8763867; doi:10.15766/mep_2374-8265.11208)
Supplement: Supplementary file 1 — Introduction to Community Organizing.pptxIntroduction to Public Narrative.pptxPredrag Stojicic Video.mp4Facilitator Manual.docxStory of Self Small-Group Guide.docxPostworkshop Survey.docx [file mep_2374-8265.11208-s001.zip › E. Story of Self Small-Group Guide.docx]

**Story of Self Small Group Guide**

### *June 8th, 2019*

CAMBRIDGE, MA

### Originally adapted from the works of Marshall Ganz of Harvard University

<http://www.hks.harvard.edu/about/faculty-staff-directory/marshall-ganz>

Modified for this training by Jonathan Shaffer and Eleanor Emery

**Originally adapted from the works of Marshall Ganz of Harvard University**

http://www.hks.harvard.edu/about/faculty-staff-directory/marshall-ganz

**Modified by the New Organizing Institute**

http://www.neworganizing.com

**Designed by Zac Willette**

ACKNOWLEDGEMENTS

We welcome your suggestions for improving this guide further for future trainings. We also welcome you to use it and adapt it for your own trainings, subject to the restrictions below.

This workshop guide has been developed over the course of many trainings by Liz Pallatto, Joy Cushman, Jake Waxman, Devon Anderson, Rachel Anderson, Adam Yalowitz, Kate Hilton, Lenore Palladino, New Organizing Institute staff, MoveOn Organizers, Center for Community Change staff, Jose Luis Morantes, Carlos Saavedra, Sean Thomas-Breitfeld, Shuya Ohno, Petra Falcon, Michele Rudy, Hope Wood, Josh Daneshforooz, Melanie Vant, Uyen Doan, Abel R. Cano, Voop de Vulpillieres and many others.

RESTRICTIONS OF USE

The following work [this workshop guide] is provided to you pursuant to the following terms and conditions. Your acceptance of the work constitutes your acceptance of these terms:

- You may reproduce and distribute the work to others for free, but you may not sell the work to others.
- You may not remove the legends from the work that provide attribution as to source (i.e., “originally adapted from the works of Marshall Ganz of Harvard University”).
- You may modify the work, provided that the attribution legends remain on the work, and provided further that you send any significant modifications or updates to [marshall_ganz@harvard.edu](mailto:marshall_ganz@harvard.edu) or Marshall Ganz, Hauser Center, Harvard Kennedy School, 79 JFK Street, Cambridge, MA 02138
- You hereby grant an irrevocable, royalty-free license to Marshall Ganz and New Organizing Institute, and their successors, heirs, licensees and assigns, to reproduce, distribute and modify the work as modified by you.
- You shall include a copy of these restrictions with all copies of the work that you distribute and you shall inform everyone to whom you distribute the work that they are subject to the restrictions and obligations set forth herein.

If you have any questions about these terms, please contact [marshall_ganz@harvard.edu](mailto:marshall_ganz@harvard.edu) or Marshall Ganz, Hauser Center, Harvard Kennedy School, 79 JFK Street, Cambridge, MA 02138

**SMALL GROUP SESSION:**

## STORY OF SELF PRACTICE WORK

**GOALS**

- Practice telling your Story of Self and get constructive feedback
- Learn to draw out and coach the stories of others

#### AGENDA

**TOTAL TIME: 45 min.**

|  | Gather in your team. Choose a **timekeeper** and **review the agenda** and **workshops**.  **Your coach will share his/her 2-minute story of self** as an example. | 5 min.  2 min. |
| --- | --- | --- |
|  | Take some time as individuals to **silently develop your “Story of Self**.”  Use the worksheet that follows. | 5 min. |
|  | As a team **go around the group** and tell your story one by one.  For each person:  - **2 minutes** to tell their story  - **3 minutes** to offer feedback from the group (use the worksheet that follows to write down your feedback)  NOTE: You have just **2 minutes to tell your story**. **Stick to this limit**. Make sure your timekeeper cuts you off. This encourages focus and makes sure everyone has a chance to tell their story. | 30 min. |
|  | **Coach invites someone** to tell their story of self to the larger group. Rejoin the larger group. | 3 min. |

**WORKSHEET:**

DEVELOPING YOUR STORY OF SELF

**Before you decide what part of your story to tell, think about these questions:**

1. Why am I called to leadership in health equity? Why am I called to this training?
2. What values move me to act? How might they inspire others to similar action?
3. What stories can I tell from my own life about specific people or events that would show (rather than tell) how I learned or acted on those values?

**What are the experiences in your life that have shaped the values that call you to leadership?**

| **FAMILY & CHILDHOOD**  Parents/Family  Growing Up Experiences  Your Community  Role Models  School | **LIFE CHOICES**  School  Career  Partner/Family  Hobbies/Interests/Talents  Experiences Finding Passion  Experiences Overcoming Challenge | **ORGANIZING EXPERIENCE**  First Experience of organizing  Connection to key books or people  Role Models |
| --- | --- | --- |

Think about the challenge, choice and outcome in your story. The outcome might be what you learned, in addition to what happened. Try drawing pictures here instead of words. Powerful stories leave your listeners with images in their minds that shape their understanding of you and your calling*.* Remember, articulating the decisions you make in the face of challenges ultimately communicates your values.

| **CHALLENGE:** | **CHOICE:** | **OUTCOME:** |
| --- | --- | --- |
|  |  |  |

**COACHING TIPS:**

**STORY OF SELF**

Remember to balance both positive and constructive critical feedback. The purpose of coaching is to listen to the way stories are told and think of ways that the storytelling could be improved.

**DON’T** simply offer vague “feel good” comments. (“That was a really great story!”)

**DO** coach each other on the following points:

- **THE CHALLENGE:** What were the specific challenges the storyteller faced? Did the storyteller paint a vivid picture of those challenges?

*“When you described ________, I got a clear picture of the challenge.”*

*“I understood the challenge to be ________. Is that what you intended?”*

*“The challenge wasn’t clear. How would you describe ________?”*

- **THE CHOICE:** Was there a clear choice that was made in response to each challenge? How did the choice make you feel? (Hopeful? Angry?)

*“To me, the choice you made was _______, and it made me feel _______.”*

*“It would be helpful if you focused on the moment you made a choice.”*

- **THE OUTCOME:** What was the specific outcome that resulted from each choice? What does that outcome teach us?

*“I understood the outcome to be _______, and it teaches me _______. But how does it relate to your work now?”*

- **THE VALUES:** Could you identify what this person’s values are and where they came from? How? How did the story make you feel?

*“Your story made me feel ________ because _________.”*

*“It’s clear from your story that you value _______; but it could be even clearer if you told a story about where that value comes from.”*

- **DETAILS:** Were there sections of the story that had especially good details or images (e.g. sights, sounds, smells, or emotions of the moment)?

*“The image of ________ really helped me identify with what you were feeling.”*

*“Try telling more details about _______ so we can imagine what you were experiencing.”*

*Coaching Your Team's “Story of Self’: As you hear each other's stories, keeping track of the details of each person’s story will help you to provide feedback and remember details about people on your team later. Use the grid below to track your team's stories in words or images.*

| **Name** | **Values** | **Challenge** | **Choice** | **Outcome** |
| --- | --- | --- | --- | --- |
|  |  |  |  |  |
|  |  |  |  |  |
|  |  |  |  |  |
|  |  |  |  |  |
|  |  |  |  |  |

*Record Feedback/Comments from Your Team Members Here:*
