# Supplementary material for: Preparing Doctors in Training for Health Activist Roles: A Cross-Institutional Community Organizing Workshop for Incoming Medical Residents
Source: MedEdPORTAL. 2022 Jan 18;18:11208. doi: 10.15766/mep_2374-8265.11208 (PMC8763867; doi:10.15766/mep_2374-8265.11208)
Supplement: Supplementary file 1 — Introduction to Community Organizing.pptxIntroduction to Public Narrative.pptxPredrag Stojicic Video.mp4Facilitator Manual.docxStory of Self Small-Group Guide.docxPostworkshop Survey.docx [file mep_2374-8265.11208-s001.zip › Educational_Summary_Report_11208.pdf]

MedEdPORTAL® | The AAMC Journal of Teaching and Learning Resources

Original Publication Open Access

Preparing Doctors in Training for Health Activist Roles: A Cross-Institutional Community Organizing Workshop for Incoming Medical Residents

Eleanor H. Emery, MD*, Jonathan D. Shaffer, Danny McCormick, MD, MPH, Jessica Zeidman, MD, Sophia R. Geffen, MPH, Predrag Stojicic, MD, MPH, Marshall Ganz, PhD, MPA, Gaurab Basu, MD, MPH

*Corresponding author: eemery@challiance.org

Abstract

Introduction: Physicians are increasingly being called on to address inequities created by social and structural determinants of health, yet few receive training in specific leadership skills that allow them to do so effectively. **Methods:** We developed a workshop to introduce incoming medical interns from all specialties at Boston-area residency programs to community organizing as a framework for effective physician advocacy. We utilized didactic sessions, video examples, and small-group practice led by trained coaches to familiarize participants with one community organizing leadership skill—public narrative—as a means of creating the relationships that underlie collective action. We offered this 3-hour, cross-institutional workshop just prior to intern orientation and evaluated it through a postworkshop survey. **Results:** In June 2019, 51 residents from 13 programs at seven academic medical centers attended this workshop. In the postworkshop survey, participants agreed with positive evaluative statements about the workshop's value and impact on their knowledge, with a mean score on all items of over 4 (5-point Likert scale, 1 = *strongly disagree*, 5 = *strongly agree*; response rate: 34 of 51). Free-text comments emphasized the workshop's effectiveness in evoking positive feelings of solidarity, community, and professional identity. **Discussion:** The workshop effectively introduced participants to community organizing and public narrative, allowed them to apply the principles of public narrative by developing their own stories of self, and demonstrated how these practices can be utilized in physician advocacy. The workshop also connected participants to their motivations for pursuing medicine and stimulated interest in more community organizing training.

Keywords
Public Narrative, Community Organizing, Physician Advocacy, Leadership Development/Skills, Anti-Racism, Diversity, Inclusion, Health Equity

Educational Objectives

By the end of this activity, learners will be able to:

1. Identify the key concepts of community organizing.
2. Identify the key concepts of one specific community organizing leadership skill: public narrative.
3. Apply the principles of public narrative by developing their own story of self.
4. Present a story of self to other learners in the session.
5. Describe one way in which public narrative skills could be utilized to support health equity work during residency training.

Citation:
Emery EH, Shaffer JD, McCormick D, et al. Preparing doctors in training for health activist roles: a cross-institutional community organizing workshop for incoming medical residents. *MedEdPORTAL*. 2022;18:11208. https://doi.org/10.15766/mep_2374-8265.11208

Introduction

Health inequities are driven in large part by social risk factors, including low socioeconomic status, lack of insurance, and disability, as well as by structural forces, including racism and discrimination on the basis of sexual orientation and gender identity.[1] Physicians are increasingly being called on to address these social and structural determinants of health and the inequities they produce.[2] Several medical professional societies and medical education thought leaders have affirmed that health advocacy aimed at addressing health inequities should be a high priority for physicians.[3-6] However, few graduate medical education programs provide training in practices and techniques that prepare residents to assume future roles as

Copyright © 2022 Emery et al. This is an open-access publication distributed under the terms of the Creative Commons Attribution license.

physician advocates for a more equitable health care system,[3,4] and few of these curricula have been published. When we reviewed curricula designed to teach advocacy skills to address health inequities in the US health care system and published in *MedEdPORTAL,*[7-13] we found that none addressed leadership development or featured community organizing, none targeted learners at the start of residency training, and few were cross-institutional.

Training in community organizing is one way physicians can learn to work with other health professionals and community stakeholders to improve health equity. Community organizing is a leadership practice that aims to enable a constituency, typically one that lacks resources and power, to identify problems they share and solutions they desire and to take collective action to achieve those solutions.[14] Harvard Kennedy School professor Dr. Marshall Ganz has developed a framework used to teach community organizing in a variety of settings, including to health professionals.[15] A key element of this framework is public narrative,[16] a storytelling process through which one develops values-based relationships with stakeholders. Training in the theory and practice of community organizing, including public narrative, could equip residents with key leadership skills needed to challenge the structural imbalances of resources that produce health inequities.

To evaluate whether training in community organizing would be a feasible and effective method to introduce residents to these key leadership skills, we developed a cross-institutional workshop targeted at incoming medical interns from all specialties at Boston-area residency programs. The workshop was designed to accomplish several objectives. First, we wanted to give participants a brief introduction to the field of community organizing with a focus on one specific leadership skill: public narrative. Second, we wanted learners to put these skills into practice by developing and delivering their own story of self. Finally, we wanted learners to consider how they could apply these practices by describing one way in which public narrative skills could be utilized to support health equity work during residency training.

Methods

Our team at the Center for Health Equity Education and Advocacy (CHEEA) at Cambridge Health Alliance (CHA), a Harvard-affiliated academic medical center in Cambridge, Massachusetts, delivered this workshop at CHA in June 2019. The workshop targeted incoming medical interns but was open to any current medical residents at the participating Boston-area residency programs.

Program Description

We designed the workshop to introduce participants to one community organizing leadership skill: public narrative. Public narrative consists of telling (1) a story of self, which communicates the values that call one to action; (2) a story of us, which identifies values shared by the teller and potential constituents that can serve as a basis for meaningful relationships and collective action; and (3) a story of now, which communicates an urgent challenge to those shared values that demands immediate action.[16] In an effort to create an efficient introductory module that could be readily incorporated into health equity and leadership development curricula, we focused this workshop on learning to craft a story of self, as this process of identifying and communicating one's values lays the groundwork for the rest of public narrative. We then situated the story of self within the broader context of public narrative and community organizing to help participants understand how the practice could be implemented. Through telling a story of self, participants also had an opportunity to reconnect with their internal motivations for pursuing health equity and, in doing so, could find inspiration for ongoing engagement in advocacy during and after residency.[4]

We modeled this workshop on Dr. Ganz's interactive framework for teaching community organizing.[14] We utilized three educational strategies: (1) interactive PowerPoint presentations to introduce the key concepts of community organizing and public narrative, (2) two video examples of public narratives followed by large-group debriefs, and (3) small groups where participants crafted their own stories of self. The small groups were overseen by coaches under the leadership of two head coaches (Jonathan D. Shaffer and Predrag Stojicic) who were alumni of Dr. Ganz's Harvard Kennedy School courses. The two facilitators who oversaw the workshop (Eleanor H. Emery and Gaurab Basu) were physicians with prior training in community organizing. Both had completed a full, 2.5-day community organizing training facilitated by Dr. Ganz and had previously coached in two or more public narrative workshops.

In planning this workshop, we gained buy-in from the participating institutions by reaching out to local residency program directors and key health equity–oriented faulty members to explain the objectives of the workshop and the utility of community organizing for residents. We worked with the program directors to select a date that preceded intern orientation for the majority of the programs so that the workshop occurred before the start of clinical activities. We recruited participants by sending an email flyer to the program directors, who then disseminated it to their incoming interns after Match

Copyright © 2022 Emery et al. This is an open-access publication distributed under the terms of the Creative Commons Attribution license.

Day. One residency program made the event a mandatory component of its intern orientation, but for all others, the event was opt-in. We did not give continuing medical education or other credit for participation.

The workshop took 3 hours and was situated within a 1-day event focused on health equity that included a keynote address on climate justice and small-group discussions on other health equity topics led by faculty from the participating programs.

The flow of the workshop is shown in Table 1. The workshop began with a brief welcome and a review of the day's agenda. We then showed an interactive PowerPoint presentation (Appendix A) featuring an overview of community organizing as a leadership practice and used the example of the 1955-1956 Montgomery bus boycott to demonstrate the importance of community organizing. This was followed by a second interactive PowerPoint presentation (Appendix B) on public narrative, which described why learning to tell a shared story is important. This presentation included two video examples, (1) a story of self, delivered by Predrag Stojicic (Appendix C), and (2) a full public narrative, delivered by James Croft (Appendix D, Suggested Resources), to demonstrate the components of a well-executed public narrative. After each example, the facilitators led a group debrief. We included speaking notes for the presentations in the respective PowerPoints (Appendices A and B) and instructions on how to integrate these presentations into the full module in the facilitator manual (Appendix D).

We then split participants into small groups where they crafted their own stories of self under the guidance of trained coaches (Appendix E, page 3). The facilitators assigned participants to small groups in advance and ensured diversity within the groups. In the groups, the coach began by sharing their own story of self and then gave all of the participants 5 minutes to brainstorm using the worksheet provided (Appendix E, page 4). Each participant then shared their story and received coaching on how to improve. At the end of the small-group session, each coach identified one member of their group to share their story in front of the large group when the full group reconvened. Finally, the facilitators led a large-group debrief soliciting feedback from the participants on highlights from the workshop and things that could be improved. They also asked participants how they imagined employing these skills in their work and gauged their interest in further training.

Table 1. Suggested Agenda

Agenda Item	Time
Welcome and introductions.	15 minutes
Introduction to community organizing.	20 minutes
Introduction to public narrative.	30 minutes
Break.	10 minutes
Introduction to story of self small groups.	10 minutes
Story of self small groups.	45 minutes
Large-group story of self sharing session.	20 minutes
Large-group discussion.	20 minutes
Closing and completion of evaluations.	10 minutes

Coach Recruiting and Training

We recruited six coaches by emailing networks of individuals who had participated in Harvard Kennedy School community organizing trainings and from participants who had participated in a similar workshop the previous year. All coaches had previously participated in at least one public narrative workshop. The two facilitators and two head coaches also served as small-group coaches for a total of 10 small groups, with a ratio of one coach to approximately five participants.

The facilitators and coaches met prior to the session for a coaching training. At the beginning of this training, the facilitators introduced the objectives and the target audience for the workshop and then reviewed the schedule for the small groups. Each coach next shared their own story of self for 2 minutes and was coached by another coach for 3 minutes using the coaching tips and worksheet in the story of self small-group guide (Appendix E, pages 5-6). The full group then offered feedback on the coaching. We rotated roles until everyone had had an opportunity to share their story and to coach. During the coaching feedback, we placed specific emphasis on ensuring that the coaches employed tactics that were positive and supportive to encourage engagement without making the participant feel forced to share information they were uncomfortable sharing. This training took approximately 90 minutes.

In preparation for this workshop, the facilitators also independently reviewed Appendices A-E, which took approximately 2 hours. Appendix D provides step-by-step instructions for conducting the workshop.

Funding and Materials

The Massachusetts General Hospital Department of Medicine Community Council funded this workshop with a grant for $2,260, which we used to reimburse the coaches for their time and to pay for participants' meals on the day of the workshop. CHEEA compensated the two facilitators via their salaries and CHA donated the physical space, which included a large room for didactic sessions and several smaller rooms for the small groups. Materials required included audiovisual equipment for showing the PowerPoint presentations and accompanying videos, a whiteboard for note-taking during the group discussions, printed

Copyright © 2022 Emery et al. This is an open-access publication distributed under the terms of the Creative Commons Attribution license.

copies of the story of self small-group guide and evaluation form, and pens.

Program Evaluation

We assessed the program's acceptability and impact on participants' attitudes towards community organizing through a postworkshop survey (Appendix F). We emailed the online survey instrument to participants 5 months after the workshop. In the survey, we asked participants to rate their level of agreement with a number of positive evaluative statements based on a 5-point Likert scale (1 = *strongly disagree,* 5 = *strongly agree*). These statements assessed the acceptability of the program as well as the degree to which it accomplished our stated objectives. We also collected qualitative data from free-text responses to questions pertaining to these objectives. To analyze these free-text responses, one author (Eleanor H. Emery) performed directed content analysis[17] that was subsequently reviewed by two additional authors (Gaurab Basu and Danny McCormick). The authors then met to discuss and resolve any discrepancies to obtain consensus.

Results

Fifty-one participants from 13 residency programs at seven academic medical centers across greater Boston attended this workshop. Of these participants, 39 (76%) were incoming interns, five (10%) were second-year residents, three (6%) were third-year residents, one (2%) was completing a chief year, two (4%) were Harvard Medical School students who assisted the organizers with coordinating the logistics for the day, and one (2%) was a doctoral student in public policy at Harvard University.

All of the positive evaluative statements, which were rated on a 5-point Likert scale (1 = *strongly disagree*, 5 = *strongly agree*), received a mean score greater than 4 (response rate: 34 of 51 participants, 67%; Table 2). The highest mean scores were for the following statements: "I am glad I attended this event" ($M = 4.4$), "This event helped me feel connected to my motivations for pursuing medicine" ($M = 4.3$), and "I think the community organizing skillset (including public narrative, story of self) is an important one for a physician to possess" ($M = 4.3$).

The free-text comments were generally consistent with the positive responses to quantitative survey items but provided greater detail regarding participants' reactions to the workshop. For example, one respondent wrote, "This type of programming is so effective in helping create a community of physicians interested in similar values." Another stated:

Table 2. Responses to Positive Evaluative Statements in the Postworkshop Survey Administered to All Participants[a]

Survey Statement[b]	*M* (*SD*)
I am glad I attended this event.	4.4 (1.0)
I would participate in a similar event in the future.	4.2 (1.2)
This event helped me feel connected to my motivations for pursuing medicine.	4.3 (0.9)
This event helped me learn how to become a more effective physician advocate.	4.0 (1.0)
I am more familiar with the concepts of community organizing (including public narrative, story of self) than I was prior to participating in this event.	4.1 (0.8)
I think the community organizing skillset (including public narrative, story of self) is an important one for a physician to possess.	4.3 (0.8)
I met trainees and faculty members at this event that I may want to collaborate with on future projects.	4.2 (0.8)

[a]Thirty-four of 51 workshop participants completed the survey, a 67% response rate.
[b]Rated on a 5-point Likert scale (1 = *strongly disagree,* 5 = *strongly agree*).

> [The workshop] was an inspiring start to the residency transition for interns and incoming juniors, too… The public narrative work continues to frame how I introduce myself on the wards and with patients; remembering I am a person and an advocate first.

Some respondents expressed concerns about keeping the group engaged in this work moving forward given clinical responsibilities, and some voiced an interest in participating in a longitudinal curriculum on community organizing after the event. Two respondents expressed concerns regarding the level of psychological vulnerability required to participate in the story of self component of the workshop. A summary of common themes that emerged in the free-text comments is shown below:

- Learning to craft a story of self was valuable as a tool for advocacy and as an opportunity to reflect on one's identity as a physician and an advocate.
- The workshop was effective in creating an opportunity to network with other trainees across hospital systems.
- The workshop generated strong feelings of community and solidarity around health equity and advocacy.
- The workshop was inspiring, and this inspiration could be carried into the start of intern year.
- The value of the workshop will depend on how well workshop participants stay engaged with one another going forward.
- Having more ongoing longitudinal community organizing trainings, more training on the practical application of these skills, and more events during the year to keep the community engaged moving forward would be valuable.
- The workshop was more about public narrative/story of self than community organizing and should be described as such in the future.

Copyright © 2022 Emery et al. This is an open-access publication distributed under the terms of the Creative Commons Attribution license.

- Discussing one's story of self in a small-group setting during a brief workshop required too much personal psychological vulnerability for the current workshop design.

Discussion

We successfully developed a cross-institutional workshop to introduce incoming medical interns to community organizing as a framework for effective physician advocacy. The evaluation responses showed that participants found the experience to be valuable and were interested in participating in more community organizing training in the future. The facilitators and coaches observed that timing the event just prior to intern orientation allowed us to capture physicians at a unique moment of reflection on their professional identities. The facilitators also found that the cross-institutional structure helped break down silos and build working relationships both amongst participants and between the workshop organizers and the participating programs.

This publication adds to the literature on the use of community organizing and public narrative to train health professionals. While Dr. Ganz's framework has been used to teach community organizing in a variety of settings, including to health professionals,[15] to our knowledge it had not yet been used as a method for training medical residents early in their careers. By targeting incoming interns, this workshop captured the unique impact that public narrative can have on professional identity for those who are still developing as physician advocates. Furthermore, while advocacy curricula are increasingly being incorporated into graduate medical education, they are not standardized, the majority are not cross-institutional, and we are not aware of any that utilize community organizing skills.[3,4] By utilizing Dr. Ganz's well-established pedagogy, this workshop features a curriculum that is readily reproducible at other centers.

Limitations

Our evaluation had several limitations. First, although we assessed participant's reactions, we did not assess any impacts on their skills or knowledge and were therefore unable to evaluate several of our educational objectives. Additionally, our sample size was small, although it did include participants from multiple academic medical centers in one city. The curriculum itself also had limitations, namely, that it was a brief, onetime intervention and that the long-term impacts of this type of isolated training on the attitudes, skills, and knowledge of participants are unknown. Many participants expressed an interest in more in-depth trainings that would cover the full spectrum of community organizing, an interest that we are working to address by developing similar modules on full public narrative and other community organizing leadership practices. To address concerns raised in the evaluation about the level of psychological vulnerability required to workshop one's story of self in front of others, we clarified in the facilitator guide that when participants are asked to share their stories in front of the large group, they should be invited to do so privately and clearly given the option to decline. We also discussed having coaches connect with their small-group participants in phone calls prior to the workshop to develop their relationship and ensure participants know what to expect in the workshop.

Future Directions

This workshop could be delivered as a stand-alone event or as part of a series of health equity–oriented modules. Though designed to target medical residents, the content is applicable across various levels of training (e.g., medical students, residents, and faculty) and health professions (e.g., physician assistants and nursing). The optimal timing for this workshop is 3 hours, but it can be shortened by sharing fewer stories in front of the large group or shortening the final discussion. The time allotted for the small groups will need to be adjusted if groups contain more than six participants. We chose to email the evaluation to participants 5 months after the workshop to gauge their reactions after completing approximately half of the intern year. However, this may have negatively impacted the response rate. To improve the response rate, we recommend conducting an initial evaluation at the end of the event, followed by a repeat evaluation partway through the year.

We are currently developing virtual modules on other community organizing leadership skills with the goal of using them sequentially to create more longitudinal learning opportunities in community organizing. We hope to conduct longer-term evaluations following these future workshops to better understand the impact on the knowledge and skills of participants. Other academic medical centers may consider the utility of implementing similar programming to provide health professionals with more opportunities to develop as effective agents of social change.

Appendices

A. Introduction to Community Organizing.pptx

B. Introduction to Public Narrative.pptx

C. Predrag Stojicic Video.mp4

D. Facilitator Manual.docx

Copyright © 2022 Emery et al. This is an open-access publication distributed under the terms of the Creative Commons Attribution license.

E. Story of Self Small-Group Guide.docx

F. Postworkshop Survey.docx

All appendices are peer reviewed as integral parts of the Original Publication.

Eleanor H. Emery, MD: Program Officer, Center for Health Equity Education and Advocacy, Cambridge Health Alliance; Instructor of Medicine, Part-time, Harvard Medical School; Medical Officer-Physician, Department of Internal Medicine, Northern Navajo Medical Center; ORCID: https://orcid.org/0000-0003-4390-0335

Jonathan D. Shaffer: Sixth-Year Doctoral Candidate, Department of Sociology, Boston University

Danny McCormick, MD, MPH: Co-Director, Center for Health Equity Education and Advocacy, Cambridge Health Alliance; Associate Professor of Medicine, Harvard Medical School

Jessica Zeidman, MD: Primary Care Program Director, Department of Internal Medicine, Massachusetts General Hospital; Instructor of Medicine, Harvard Medical School

Sophia R. Geffen, MPH: Program Manager, Center for Health Equity Education and Advocacy, Cambridge Health Alliance

Predrag Stojicic, MD, MPH: Instructor, Harvard T.H. Chan School of Public Health; Program Director for Community Organizing, Center for Health Equity Education and Advocacy, Cambridge Health Alliance; Executive Director, People Power Health

Marshall Ganz, PhD, MPA: Rita E. Hauser Senior Lecturer in Leadership, Organizing, and Civil Society, Harvard Kennedy School

Gaurab Basu, MD, MPH: Co-Director, Center for Health Equity Education and Advocacy, Cambridge Health Alliance; Instructor of Medicine, Harvard Medical School

Disclosures
None to report.

Funding/Support
The Massachusetts General Hospital Department of Medicine Community Council funded this workshop with a grant for $2,260.

Ethical Approval
The Cambridge Health Alliance Institutional Review Board deemed further review of this project not necessary.

References

1. Phelan JC, Link BG, Tehranifar P. Social conditions as fundamental causes of health inequalities: theory, evidence, and policy implications. *J Health Soc Behav*. 2010;51(1)(suppl): S28-S40. https://doi.org/10.1177/0022146510383498
2. Metzl JM, Hansen H. Structural competency: theorizing a new medical engagement with stigma and inequality. *Soc Sci Med*. 2014;103:126-133. https://doi.org/10.1016/j.socscimed.2013.06.032
3. Howell BA, Kristal RB, Whitmire LR, Gentry M, Rabin TL, Rosenbaum J. A systematic review of advocacy curricula in graduate medical education. *J Gen Intern Med*. 2019;34(11): 2592-2601. https://doi.org/10.1007/s11606-019-05184-3
4. McDonald M, Lavelle C, Wen M, Sherbino J, Hulme J. The state of health advocacy training in postgraduate medical education: a scoping review. *Med Educ*. 2019;53(12):1209-1220. https://doi.org/10.1111/medu.13929
5. Dharamsi S, Ho A, Spadafora SM, Woollard R. The physician as health advocate: translating the quest for social responsibility into medical education and practice. *Acad Med*. 2011;86(9): 1108-1113. https://doi.org/10.1097/ACM.0b013e318226b43b
6. Basu G, Pels RJ, Stark RL, Jain P, Bor DH, McCormick D. Training internal medicine residents in social medicine and research-based health advocacy: a novel, in-depth curriculum. *Acad Med*. 2017;92(4):515-520. https://doi.org/10.1097/ACM.0000000000001580
7. Nehal US, Kanahara S, Tanabe M, Hayner G, Nelson BD. Pediatric refugee health care delivery in the community setting: an educational workshop for multidisciplinary family-centered care during resettlement. *MedEdPORTAL*. 2020;16:10988. https://doi.org/10.15766/mep_2374-8265.10988
8. McDonald M, West J, Israel T. From identification to advocacy: a module for teaching social determinants of health. *MedEdPORTAL*. 2015;11:10266. https://doi.org/10.15766/mep_2374-8265.10266
9. Chung EK, Kahn S, Altshuler M, Lane JL, Plumb J. The JeffSTARS Advocacy and Community Partnership Elective: a closer look at child health advocacy in action. *MedEdPORTAL*. 2016;12:10526. https://doi.org/10.15766/mep_2374-8265.10526
10. Hoffman BD, Rose J, Best D, et al. The Community Pediatrics Training Initiative project planning tool: a practical approach to community-based advocacy. *MedEdPORTAL*. 2017;13:10630. https://doi.org/10.15766/mep_2374-8265.10630
11. Majeed A, Newton H, Mahesan A, Vazifedan T, Ramirez D. Advancing advocacy: implementation of a child health advocacy curriculum in a pediatrics residency program. *MedEdPORTAL*. 2020;16:10882. https://doi.org/10.15766/mep_2374-8265.10882
12. Marsh MC, Supples S, McLaurin-Jiang S, Brown CL, Linton JM. Introducing the concepts of advocacy and social determinants of health within the pediatric clerkship. *MedEdPORTAL*. 2019;15: 10798. https://doi.org/10.15766/mep_2374-8265.10798
13. Sola O, Kothari P, Mason HRC, Onumah CM, Sánchez JP. The crossroads of health policy and academic medicine: an early introduction to health policy skills to facilitate change. *MedEdPORTAL*. 2019;15:10827. https://doi.org/10.15766/mep_2374-8265.10827

Copyright © 2022 Emery et al. This is an open-access publication distributed under the terms of the Creative Commons Attribution license.

14. Ganz M. Leading change: leadership, organization, and social movements. In: Nohria N, Khurana R, eds. *Handbook of Leadership Theory and Practice: A Harvard Business School Centennial Colloquium*. Harvard Business Press; 2010:527-568.
15. Jabbar A, Molnar F, Sinha S. Advocating for improved health care for older Canadians: what we can learn from applying the Marshall Ganz Advocacy Framework. *Can Geriatr J CME.* 2019;9(1):11-18.
16. Ganz M. Public narrative, collective action, and power. In: Odugbemi S, Lee T, eds. *Accountability Through Public Opinion: From Inertia to Public Action*. World Bank; 2011:273-289. https://doi.org/10.1596/9780821385050_CH18
17. Hsieh HF, Shannon SE. Three approaches to qualitative content analysis. *Qual Health Res.* 2005;15(9):1277-1288. https://doi.org/10.1177/1049732305276687

Received: March 9, 2021
Accepted: September 22, 2021
Published: January 18, 2022

Copyright © 2022 Emery et al. This is an open-access publication distributed under the terms of the Creative Commons Attribution license.
